# Assessing grid affordability for universal electricity access in Sub-Saharan Africa

**DOI:** 10.1038/s41467-026-75104-7

**Published:** 2026-07-09

**Authors:** Yidan Kou, Stephanie Hirmer, Pu Yang, Alycia Leonard, Florian Egli

**Affiliations:** 1 Department of Engineering Science, University of Oxford, Oxford, UK; 2 Institute of Sustainable Resources, The Bartlett School of Environment, Energy and Resources, University College London, London, UK; 3 School of Social Sciences and Technology, Technical University of Munich, Munich, Germany; 4 School of Management, Technical University of Munich, Munich, Germany

**Keywords:** Energy access, Energy supply and demand, Energy economics, Social sciences

## Abstract

Grid expansion remains a key strategy for increasing electricity access across Sub-Saharan Africa (SSA). However, whether utilities can generate sufficient revenue under current tariffs to support capital investment is unclear. We compile a comprehensive dataset of residential electricity tariffs for 48 SSA countries and develop a standardized model to estimate electricity bills using the Multi-Tier Framework (MTF). Affordability is assessed across income percentiles using simulated income distributions. Under current tariffs, around 608 million people (50%) may not afford Tier 4 electricity, rising to 1.05 billion (85%) for Tier 5 under a 10% energy-poverty threshold. Sensitivity analyses using 5% and 15% thresholds confirm the robustness of these findings. Even where higher-tier electricity is affordable, usage remains low. Combining affordability estimates with utility financial performance, we identify where grid expansion could be viable and where affordability constraints and utility deficits can create electrification traps. In many countries, off-grid solutions may be the only feasible pathway to achieving SDG7.

## Abstract (web summary)

This study compiles a region-wide database of residential electricity tariffs in Sub-Saharan Africa and shows that current grid expansion strategies may create electrification traps, leaving electricity unaffordable for households and financially unsustainable for utilities.

## Introduction

Expanding electricity grids is seen as essential for achieving ‘universal access to affordable, reliable and modern energy services’ by 2030 as stipulated in Sustainable Development Goal 7.1^1^, because it remains the most scalable and long-term pathway for mass electrification in Sub-Saharan Africa (SSA). Yet, while many countries have relied on grid expansion as their primary electrification strategy^2,3^, progress using this approach has been slow. The reported number of people without access to electricity in the region decreased only marginally from 614 to 584 million between 2015 and 2021^4^.

Grid expansion requires capital investments from national utilities. However, many SSA utilities face major financial struggles. According to 2024 data, 63% of power utilities in SSA operated at a loss over the three most recent years, with deficits exceeding 80% of total revenues in São Tomé and Príncipe, Angola, and Central African Republic^5^. For utilities to achieve or maintain financial sustainability, they need to generate adequate revenue from customers. This requires a customer base that is both connected to the grid and consuming sufficient levels of electricity. Empirically, however, we observe issues in both.

First, households often remain disconnected from the grid even though access is technically feasible^6–8^. For example, in Kenya, a study showed that although the electricity grid covered 74% of households in the surveyed areas, only 54% had a physical connection^9^. This gap is even more pronounced in rural areas^10^. Low connection rates often stem from affordability constraints, as power grids in SSA impose high connection fees and tariffs that rank among the highest in the world^11,12^.

Second, even after gaining grid access, electricity usage has remained low in countries such as Rwanda^13^, Tanzania^14^, and South Africa^15^, largely because grid electricity is unaffordable^9^. In some settings, low reliability further discourages electricity use^16–18^. Even worse, arbitrary or unsubstantiated tariff-setting may drive high-consuming customers off-grid (e.g. farms and agribusinesses in South Africa^19^). To better inform grid expansion decisions, we need evidence on whether households with different income and consumption levels can afford electricity bills under existing tariff structures across SSA^6^.

Here, we assess the viability of grid expansion to increase electricity access under current residential tariff structures in 48 SSA countries. We focus on grid-based electrification because most countries still treat it as their primary strategy, and because data availability constrains comparable analysis of off-grid solutions. The grid retains clear advantages in densely populated areas with higher demand, where economies of scale apply. These advantages weaken for last-mile connections in remote or sparsely populated regions, where off-grid solutions remain critical and may be the more effective pathway to universal access.

Using the Multi-Tier Framework for Measuring Energy Access (MTF)^20^, we evaluate the affordability of residential electricity at different demand levels (i.e., tiers). To do so, we compile a comprehensive dataset of residential electricity tariffs for each SSA country and use this to estimate electricity bills by tiers. As expanding access requires high capital expenditure to reach more remote populations, we use current tariffs to provide a maximally optimistic estimate of affordability. Based on estimated income distributions by country, we calculated the share of the population able to afford prevailing grid electricity tariffs under different demand levels. We find that affordability declines substantially at higher consumption tiers, constraining electricity consumption for large segments of the population in many SSA countries. The results are juxtaposed with the financial health of utilities to systematically map potential opportunities and pitfalls when trying to reach electrification goals via grid expansion.

## Results

### Grid tariffs and electricity bills

**Fig. 1:**
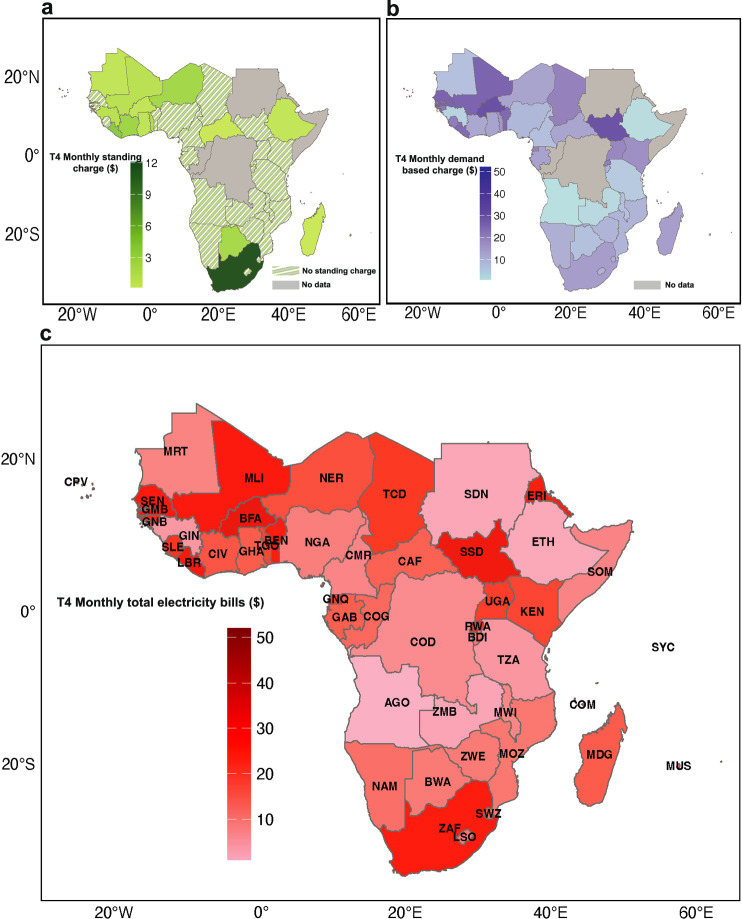
T4 Monthly residential electricity bills across SSA countries in 2024 USD ($). **a** Monthly standing charges (fixed monthly fees paid by households). Green hatched areas indicate countries without standing charges. **b** Monthly demand-based charges based on electricity consumption (kWh), active power (kW), apparent power (kVA) and current (A), including applicable taxes. **c** Total electricity bills derived from (**a**) and (**b**). For countries with missing data in (**a**) and (**b**) (grey areas), total bills in (**c**) were estimated using average residential electricity bills. All underlying data are available on Zenodo in Summary_Electricity_Bills.xlsx. African administrative boundaries were obtained from Esri ArcGIS^57^. Countries are labelled using ISO alpha-3 country codes.

Monthly electricity bills are first estimated for each country in SSA separately, based on the energy usage of Tier 4 (T4) (i.e., 102 kWh per month—see ‘Methods’ for details). T4 is assumed to be the lowest consumption level that would justify grid expansion as opposed to off-grid electrification^20^. We provide results for Tier 3 (T3) and Tier 5 (T5) in the Supplementary Information as lower- and upper-bound sensitivity cases. All results are discussed in US dollars ($). Electricity bills across SSA countries exhibit large variance, as shown in Fig. 1. We find that monthly residential electricity bills at T4 range from as low as $1 in Angola to a staggering $52 in Comoros. Bills range from $0.13 to $15 for T3 and escalate to $7 to $125 for T5 (see Supplementary Figs. 1 and 2), highlighting that electricity bills rise sharply with higher consumption levels.

Moreover, Fig. 1 highlights distinct regional patterns. Geographically adjacent countries such as Angola, Zambia, and Democratic Republic of the Congo show low bills, averaging around $5 per month. In contrast, neighbouring countries in West Africa—Mali, Burkina Faso, and Senegal—exhibit much higher bills, averaging around $30 per month. These regional disparities may reflect differences in production costs, market structures, and cross-border power-pool dynamics.

We also find that electricity bills are largely driven by demand-based charges (Fig. 1b), which vary based on how much electricity a household consumes, rather than by standing charges (Fig. 1a), which are fixed fees that households pay regardless of their consumption. Among the 48 countries analysed, 27 impose no standing charges, and most of the remaining countries set standing charges below $6 per month. South Africa is the notable exception, with a relatively high standing charge of $12 per month^21,22^. This indicates that, in most SSA countries, connection-related costs are either subsidised or excluded from monthly cost recovery; however, electricity remains expensive due to high demand-based tariffs.

**Fig. 2:**
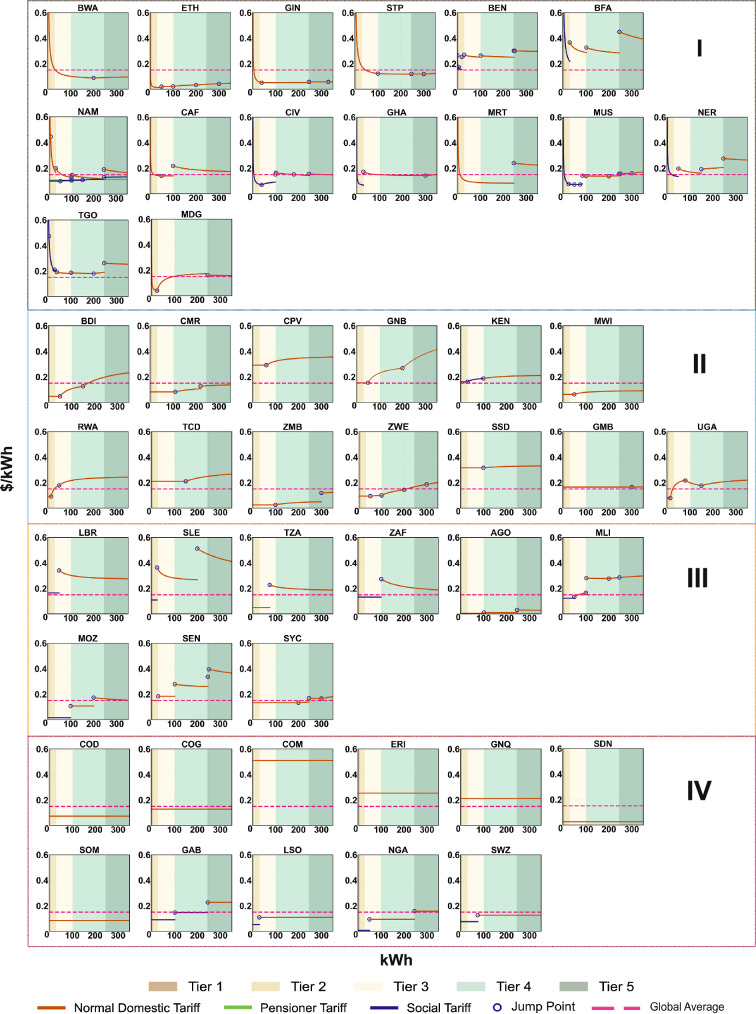
Residential electricity unit prices ($/kWh) at different monthly consumption levels across SSA countries. Countries are grouped into four categories (I–IV) according to how tariff structures shape effective unit prices as consumption increases. The unit price in each panel was calculated by dividing the total monthly electricity bill by the corresponding monthly consumption level. Background shading shows the corresponding MTF electricity access tiers (T1–T5). For the Democratic Republic of the Congo (COD), Republic of the Congo (COG), Eritrea (ERI), Somalia (SOM), and Sudan (SDN), only average price were available. Detailed residential tariff structures are shown in Supplementary Table 1. Countries are labelled using ISO alpha-3 codes.

Residential electricity bills in SSA are determined not only by the distinction between standing and demand-based charges, but also by tariff band structures and targeted mechanisms such as social tariffs. While Fig. 1 decomposes bills into standing charges and demand-based components, Fig. 2 indicates how the average unit price ($/kWh), inclusive of both components, varies with electricity consumption across countries under different residential tariff structures, suggesting four distinct patterns (Groups I–IV).

Countries in Group I (15 countries) impose consumption-independent standing charges, resulting in high average unit prices at low consumption levels (T1–T2) and disproportionately burdening poorer households. This regressive effect persists even among the eight countries within this group (e.g. Benin, Burkina Faso, and Côte d’Ivoire) that operate social/lifeline tariffs intended to support low-income and vulnerable households. Because standing charges apply under the social/lifeline tariff as well, households consuming less than 30 kWh per month still face higher effective per-kWh bills than higher-consuming households on standard tariffs.

Countries in Group II (13 countries) apply increasing block tariffs (IBTs) without standing charges. In these countries, average unit prices increase progressively across consumption levels, although the steepness of the tariff structure varies widely depending on block thresholds and corresponding prices. Although most countries in this group—aside from Kenya and Uganda—do not implement explicit social/lifeline tariffs, the IBT structure itself offers lower effective prices for low-consumption households. When block increments are moderate, households can transition more smoothly to higher consumption levels; however, steep marginal price increases may render additional consumption unaffordable, suppress demand, and in some cases encourage households to disconnect from the grid. At one extreme, households in Guinea-Bissau pay $0.15/kWh in Tier 1 (T1) and $0.40/kWh in T5; by contrast, in Kenya, Malawi, and Gambia, the increase from T1 to T5 is less than $0.05/kWh.

Countries in Group III (9 countries), five of which have social/lifeline tariffs, exhibit patterns similar to those in Group II. However, the combination of IBTs and standing charges produces more abrupt price increases at specific consumption thresholds. This leads to elevated effective unit prices at the lower end of each tariff band. For example, in Sierra Leone, the unit price triples from $0.11/kWh to $0.35/kWh once monthly electricity consumption exceeds 25 kWh. As a result, marginal increases in electricity consumption at tariff thresholds can trigger disproportionately large increases in effective residential electricity prices.

Countries in Group IV (11 countries) set linear tariffs without standing charges. Four countries in this group implement social/lifeline tariffs (e.g., Gabon and Lesotho), while the others apply uniform linear tariffs across all consumption levels. Without standing charges or block thresholds, the effective unit price does not vary with consumption, so affordability outcomes depend on the level at which the linear tariff is set rather than on the structure itself.

Across all groups, Fig. 2 shows that unit prices at T4 and T5 exceed the global average (i.e., $0.15/kWh in Q4 2024^23^) in more than half of SSA countries. Notably, in Benin, Burkina Faso, Cape Verde, Togo, and South Sudan, electricity prices are two to three times higher than the global average. In these predominantly low-income countries, such high price levels substantially limit the ability of households to increase electricity consumption and progress toward higher MTF tiers, a key objective of SDG7. While the MTF tiers are used here as a comparative benchmark, owing to their role in SDG7 monitoring^20^, they do not necessarily correspond to national tariff blocks. Nevertheless, they provide a useful reference framework for evaluating affordability across different levels of electricity consumption. Overall, the substantial cross-country variation in tariff levels and structures, combined with generally high unit prices at moderate and high consumption levels, suggests that affordability remains a major barrier to meaningful electricity use across the region.

The extent to which these tariffs constrain electricity use depends on how residential bills compare with household incomes. When bills exceed what households can afford, electricity demand and grid connection rates are suppressed. Lower consumption, in turn, reduces utility revenues and limits the financial capacity of utilities to maintain reliable supply and extend the grid. We examine these three linked questions in turn: (1) residential affordability across income percentiles, (2) the gap between affordability and realised demand, and (3) the financial position of utilities at prevailing tariffs, which we disentangled in the sections below.

### Affordability

To evaluate the affordability of grid electricity, we construct income distributions for each country by fitting a lognormal model to population, GDP per capita, and the Gini index, and then compare the resulting income percentiles with the monthly residential electricity bill for T4 and T5 consumption levels (see ‘Methods’). Households at a given income percentile are classified as unable to afford electricity when the required bill exceeds 10% of the income at that percentile, with 5% and 15% used as sensitivity bounds. We adopt 10% as the primary threshold because it is widely used to define energy poverty^14,15,24–28^. Under this threshold, approximately 50% of the SSA population cannot afford T4 electricity, rising to 85% for T5. Sensitivity bounds give 71% and 38% at T4 (5% and 15% thresholds), and 97% and 76% at T5 (Supplementary Table 2). Although absolute affordability levels vary across thresholds, country rankings remain broadly consistent under the 5%, 10%, and 15% thresholds.

**Fig. 3:**
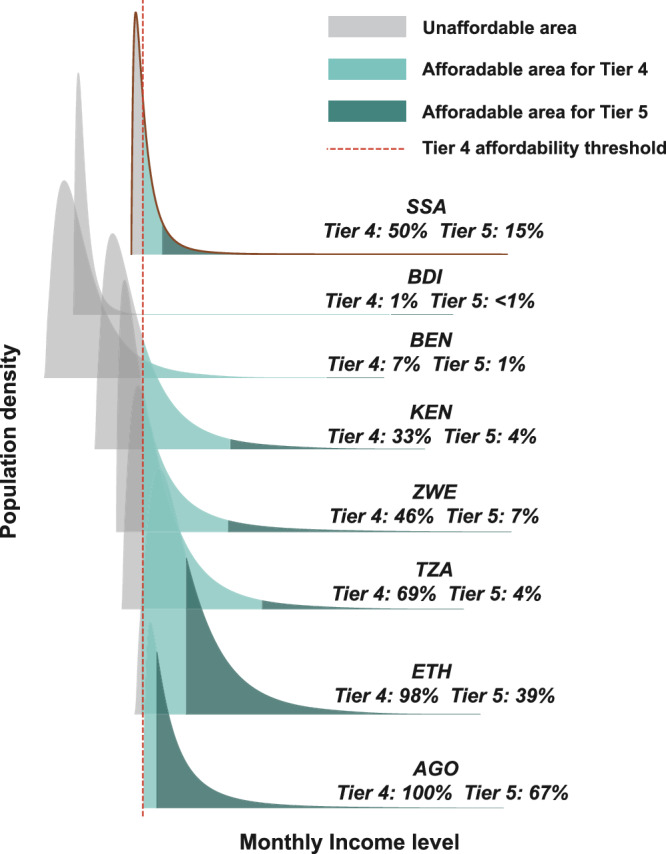
Income distributions by country and the proportion of the population able to afford T4 and T5 electricity access. The first curve shows the aggregated population-weighted income distribution in SSA. The remaining curves present seven illustrative countries spanning a range of electricity affordability outcomes. Each distribution is estimated using kernel density estimation (KDE) in log-income space and transformed to linear scale via Jacobian correction. The x-axis shows monthly income (USD/month) and the y-axis population density. Different colours indicate the affordability of T4 and T5 electricity bills under the 10% income rule. Detailed results for all 48 countries under 5%, 10%, and 15% affordability thresholds are provided in Supplementary Table 2. Countries are labelled using ISO alpha-3 codes.

Figure 3 presents the aggregated income distribution for SSA along with the percentage of the population capable of obtaining electricity access to T4 and T5 under 10% energy-poverty threshold. It also includes seven illustrative country cases to highlight how affordability outcomes vary according to where electricity bill levels intersect with each country’s income distribution, ranging from the lowest affordability in Burundi to the highest in Angola. Detailed results are provided in Supplementary Table 2. Although the shapes of the distributions differ, the key driver of affordability is the relative position of the T4 and T5 bill levels within these distributions.

We find that approximately 608 million people (50% of the total population in SSA) may not afford the minimum electricity consumption required to achieve access to T4 under the 10% poverty line. Affordability constraints are particularly severe in countries such as Burkina Faso, Burundi, Central African Republic, Chad, Comoros, Eritrea, Guinea-Bissau, Liberia, Madagascar, Mali, Niger, and Sierra Leone, where fewer than 10% of the population can afford T4-level electricity access at the 10% benchmark. By contrast, in countries such as Angola, Mauritius, Seychelles, over 90% of the population are theoretically able to cover the associated electricity bills (Supplementary Table 2). At the T5 level, affordability deteriorates sharply: approximately 1.05 billion people, representing 85% of the SSA population, are unable to afford the required electricity expenditure. In Mauritania, affordability declines by 82 percentage points, leaving only 2% of the population able to afford T5 electricity access. Similar declines are observed in Nigeria (66%), Tanzania (65%), and Guinea (62%), as shown in Supplementary Table 2. These findings underscore that higher tier electrification via grid access is beyond reach for most households under current income levels.

Cross-country differences in affordability are shaped by both income levels and electricity tariffs, although income disparities account for much of the variation observed across SSA. This pattern is illustrated by contrast between Burundi and Zimbabwe: although similar T4 and T5 electricity bills ($0.10–$0.30 per kWh; see Figs. 1, 2), affordability outcomes differ strikingly. In Burundi, only the wealthiest 1% of the population (0.14 million) can afford the bills associated with T4 and T5 electricity consumption. By contrast, affordability is far more widespread in Zimbabwe, where an estimated 46% of the population (7.65 million) can afford T4, and 7% (1.16 million) can afford T5. This disparity reflects the large income gap, with average monthly per capita income at $145 in Zimbabwe versus just $20 in Burundi (see ‘Data availability’). Conversely, countries with comparable low-income levels can display very different affordability outcomes when tariff levels diverge. For example, Angola and Comoros have similar incomes among the lowest decile, but Comoros’ T4 bill ($52) is more than fifty times higher than Angola’s ($1), shown in Fig. 1. As a result, T4 access is effectively unaffordable for low-income households in Comoros while remaining easily attainable in Angola. These contrasts suggest that tariff levels may exert their strongest influence at the lower end of the income distribution, where even modest price differences can completely exclude low-income households from accessing higher electricity tiers. Overall, national affordability profiles are primarily shaped by broader economic capacity, whereas affordability differences across income groups are strongly influenced by tariff structures.

This asymmetry is further reflected in the Group I countries in Fig. 2. With the exception of Mauritius, standing charges raise the effective unit price of very low consumption levels (T1–T2) to levels that are already unaffordable for most low-income households. Although the effective unit price declines as consumption increases toward T4 and T5—a pattern observed across all tariff structures—affordability simulations indicate that these households remain unable to afford the resulting total monthly electricity expenditure, even under optimistic assumptions. In practice, low-income households are therefore caught between prohibitively high unit prices at minimal levels of consumption and unaffordable total bills at higher consumption levels, rendering grid electricity effectively out of reach. Consequently, despite the existence of nominal social/lifeline tariffs in several countries, prevailing tariff structures fail to provide meaningful protection for low-income users and do not facilitate progression beyond minimal levels of electricity access.

### Demand expectations

**Fig. 4:**
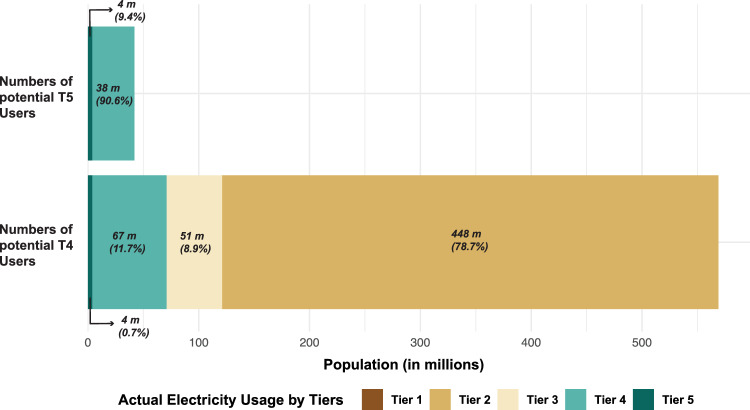
Comparison between theoretical electricity affordability and observed residential electricity consumption. Each country is assigned to an observed MTF energy tier based on national average residential electricity consumption, and total population is aggregated accordingly. Bars show the population distribution across tiers for the 13 countries where T4 or T5 electricity access is theoretically affordable under the 10% income rule. Detailed country-level results for affordability and electricity consumption are provided in Supplementary Tables 3 and 4.

Our analysis suggests that electricity affordability does not always translate into high actual demand, as illustrated in Fig. 4. To examine this relationship, we compare country-average electricity affordability with observed electricity demand using data from the International Energy Agency (IEA) and Index Mundi (see ‘Methods’). Due to data availability, the analysis is conducted at the national level, with corresponding electricity access tiers identified accordingly. This allows us to assess whether countries with the financial capacity to afford T4 and T5 levels of electricity consumption achieve these consumption levels in practice.

We find that affordability is a necessary but not sufficient condition for achieving higher-tier electricity consumption. Even in settings where T4 and T5 electricity is theoretically affordable, actual household demand often falls short. For example, among countries where T4 electricity is considered affordable on average, 88% of the population—equivalent to approximately 499 million people—do not attain T4 consumption levels in practice. Specifically, in Ethiopia, Guinea, and Tanzania, where households can theoretically afford T4 electricity access, average monthly residential electricity demand remains at only 21 kWh, 29 kWh, and 18 kWh, respectively, corresponding to Tier 2 (T2) (see Supplementary Tables 3 and 4). Furthermore, among the four countries where T5 electricity is generally affordable, only Botswana, Mauritius, and Seychelles exhibit a clear alignment between affordability and actual demand, together representing a population of approximately 4 million people. In the fourth country, Angola, average monthly residential electricity consumption is approximately 128 kWh, corresponding to mid-T4 rather than T5 (see Supplementary Tables 3 and 4).

These discrepancies highlight that once affordability barriers are removed, supply-side constraints become the binding limitation, such as grid reliability. Nigeria provides a notable example: despite households spending only 8.5% of income on electricity (see Supplementary Table 3) and around 70% of the population having access to electricity^7^, frequent voltage collapses hinder consistent electricity use^29^. From 2010 to 2023, the national grid has experienced approximately 222 instances of partial or total collapse, leading to recurrent blackouts and undermining the stability of electricity supply^30^. This underscores the need for granular, country-specific analyses of supply-side bottlenecks to support meaningful electricity access.

### Balancing utility health and tariff affordability

**Fig. 5:**
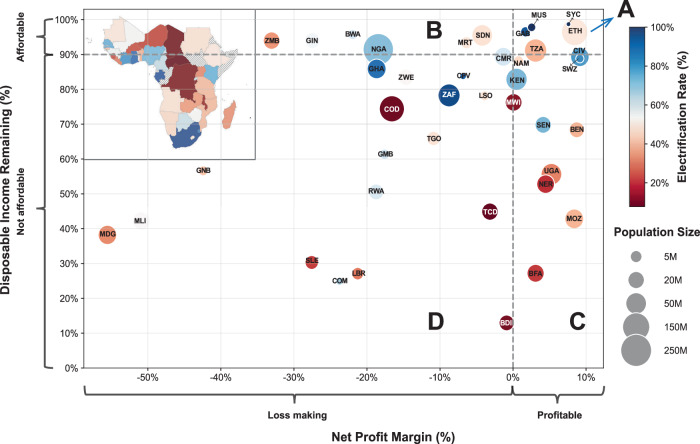
Utility financial health and T4 residential electricity affordability comparison. The x-axis shows the net profit margin of distribution utilities or vertically integrated utilities (VIUs), used as an indicator of utility financial health, with data obtained from UPBEAT^5^. The y-axis shows the percentage of household income remaining after T4 electricity expenditure (102 kWh/month). The dashed horizontal line at 90% represents the affordability threshold, indicating that electricity expenditure does not exceed 10% of household income. And the dashed vertical line at zero net profit margin represents the financial health threshold. Within this framework, the four quadrants illustrate different electricity access conditions and challenges: the upper-right quadrant (**A**) represents grid electrification opportunity; the upper-left quadrant (**B**) indicates utility stress; the lower-right quadrant (**C**) reflects affordability stress; and the lower-left quadrant (**D**) represents a grid electrification trap. Bubble size represents population and colour indicates national electrification rate. Net profit margin data were unavailable for Equatorial Guinea (GNQ), Eritrea (ERI), Republic of the Congo (COG), Somalia (SOM), and South Sudan (SSD), which are indicated by grey parallel lines in the inset African map. Angola (AGO), Central African Republic (CAF), and São Tomé and Príncipe (STP) are excluded as outliers. Complete Figure is provided in Supplementary Fig. 4. African administrative boundaries were obtained from Esri ArcGIS^57^. Countries are labelled using ISO alpha-3 codes.

Figure 5 indicates how the feasibility of grid expansion jointly depends on residential electricity affordability under T4 consumption and the financial health of national utilities, leading to distinct electrification pathways across SSA. Corresponding results for T3 and T5 are presented in Supplementary Figs. 3 and 5, respectively.

First, countries in the grid electrification opportunity quadrant (A) combine strong residential electricity affordability with solvent utilities, yet still contain approximately 110 million unelectrified people. Expanding the grid could bring substantial benefits to underserved region. However, although countries in quadrant A exhibit strong potential for grid expansion, the spatial distribution of unelectrified households remains a critical consideration for implementation. In Gabon, households spend, on average, only 3% of their income to afford T4 electricity access, while the national utility, Société d’Énergie et d’Eau du Gabon (SEEG), maintains a profit margin equivalent to 1.7% of revenue. These findings suggest that the utility possesses sufficient financial capacity to support grid expansion and that households are generally able to afford electricity at prevailing tariff levels. Despite this favourable combination, electrification remains highly uneven. By 2023, although Gabon had achieved a national electrification rate of 94%^31^, rural areas lagged substantially behind, with only 29% coverage^32^. Rural populations are widely dispersed across tropical rainforests and hilly terrains, whereas electricity generation and transmission infrastructure is concentrated in coastal and central urban centres such as Libreville, Port-Gentil, and Franceville. This spatial mismatch reduces the economic viability of grid expansion to remote rural areas. Similar rural-urban disparities in Ethiopia and Tanzania indicate that affordability and utility solvency are primarily supported by urban customers^31–33^, whereas rural electrification remains economically challenging without cross-subsidisation from urban revenues or dedicated public support. By contrast, in smaller island states such as Mauritius and Seychelles, grid expansion can more readily deliver last-mile electrification.

Second, countries in utility stress (B) and affordability stress (C) face at least one major barrier to grid expansion. In the affordability stress quadrant, countries such as Senegal and Uganda maintain financially viable power utilities; however, high electricity prices constrain residential consumption and limit new connections to the grid. In contrast, in the utility stress quadrant, countries such as São Tomé and Príncipe, and Angola (see Supplementary Fig. 4), exhibit relatively strong affordability; yet, their electricity providers incur financial losses amounting to 1.75 times and 0.88 times their revenue, respectively. Such conditions may result from inadequate cost recovery, low utility management efficiency, high generation costs, and unsustainable tariff structures, thereby constraining utilities’ ability to finance even basic system maintenance, let alone grid expansion^34^.

Finally, countries in the grid electrification trap quadrant (D)—such as Madagascar and Sierra Leone—experience both utility deficits and low household purchasing power. Approximately 220 million people in this quadrant are unlikely to gain access to affordable grid-based electricity in the short term. A particularly severe example is Central African Republic, where the national utility incurs financial losses equivalent to 87% of its revenues, and households would need 65% of their income to afford electricity (see Supplementary Fig. 4). In Central African Republic, only around 0.3 million people (6% of the population) currently have access to electricity^7^, while approximately 60% of the population resides in rural areas^35^. The combination of limited grid infrastructure, a weak national utility, spatially dispersed households, and political instability makes large-scale grid expansion neither technically feasible nor financially viable in the short term^35^. In such contexts, off-grid or mini-grid solutions, particularly through Public Private Partnership (PPP), may offer a more viable path to electrification. Under models in which governments retain ownership of infrastructure while private operators manage system operation and maintenance, capital and operational responsibilities can be shared more effectively. Such arrangements may reduce the financial burden on public utilities while improving the stability and reliability of electricity supply.

## Discussion

To examine the reasons behind the current grid electrification status in SSA, this study provides a macro-level evaluation of residential affordability under existing bills in different MTF energy tiers. We developed the most comprehensive electricity tariff database for SSA to date, covering both standing and demand-based charges. Our findings indicate that grid electrification progess are shaped by the interaction between residential electricity unaffordability, suppressed electricity demand, and utility underfunding.

Taken together, affordability challenges arise not only from low incomes but also from tariff structures that systematically constrain low-income households from progressing beyond minimal levels of electricity consumption. Even where social/lifeline tariffs are in place, prevailing tariff designs often fail to deliver effective affordability, both at existing levels of electricity use and in supporting sustained progression toward higher energy tiers. This disconnect underscores the urgent need for several SSA countries—particularly those in Group I shown in Fig. 2—to reassess the tariff design and targeting mechanisms to ensure that cost recovery objectives can be achieved without trapping low-income households in persistently low levels of electricity consumption.

However, even where grid electricity is theoretically affordable, actual consumption often remains far below potential (see Fig. 4 and Supplementary Table 4). On the demand side, low electricity consumption is driven in part by limited household ability to afford appliances required for higher-tier electricity use^36,37^, as well as continued reliance on traditional fuels such as charcoal^38^. On the supply side, underfunded utilities frequently struggle to recover costs and to maintain or expand grid infrastructure (see Fig. 5). The resulting service unreliability—manifested in frequent outages, voltage instability, and, in some cases, large-scale grid collapses^39^—further suppresses residential electricity use, reinforcing a persistent electrification trap. Together, these findings suggest that expanding grid access alone is unlikely to deliver meaningful electrification outcomes unless affordability, appliance access, and service reliability constraints are addressed.

Against this backdrop, grid expansion must be carefully assessed, as it is not always practical or economically viable. Extending services into low-affordability areas without complementary demand-side support—such as connection subsidies or appliance financing schemes—may impose additional fiscal pressure on utilities and reinforce patterns of underutilisation and declining investment (the grid electrification trap quadrant in Fig. 5). Governments should therefore give careful consideration to when and where grid electrification is viable under prevailing tariffs. In many cases, although beyond the primary scope of this paper, off-grid solutions may offer a more viable alternative. Such approaches have already been successfully implemented in several SSA countries, including Tanzania, Mali, and Ghana^40,41^.

Nevertheless, several sources of uncertainty should be acknowledged. Income distributions were modelled using lognormal functions calibrated to GNI per capita and the Gini coefficient, which may not fully capture the multimodality or heavy upper tails observed in empirical income data, thereby introducing uncertainty into affordability estimates. In addition, the assessment of utility financial performance considers only residential tariff revenues and does not fully account for cross-subsidisation from industrial, commercial, and public-sector customers. Finally, residential demand was approximated using observed consumption data because consistent demand datasets remain unavailable for many SSA countries. Observed consumption may therefore underestimate latent demand under affordability or reliability constraints.

Despite these limitations, the analysis highlights how affordability constraints, suppressed electricity demand, and utility underfunding can interact to reinforce persistent electrification challenges across SSA. More fundamentally, the findings suggest that the success of electrification strategies should not be evaluated solely by the number of grid connections achieved, but by whether electricity services are affordable, reliable, and capable of supporting meaningful residential electricity use. Achieving this will require electrification strategies that are better aligned with local demand conditions and institutional realities, supported by measures such as appliance financing, targeted tariff subsidies, selective utility reform, and, where appropriate, off-grid alternatives. Such approaches will likely be essential for avoiding the electrification trap and achieving equitable and sustainable energy access across SSA.

## Methods

Our methodology offers a transparent and comparable framework to assessing residential electricity affordability across population percentiles in 48 countries, despite limited data availability. We rely on country-specific residential tariff structures and model income distributions using widely applied approaches (e.g., log-normal distribution) to ensure consistency. Although the modelled distributions inevitably simplify some within-country heterogeneity, they enable the identification of broad regional patterns and the assessment of affordability across energy tiers for different income groups in a manner that is both robust and replicable.

### Building a comprehensive tariff database for SSA

Residential electricity tariffs were collected for 48 countries. Tariffs effective on 15 November 2024 were obtained from official utility documents for 38 countries. For Angola, Burundi, Chad, Comoros, and Equatorial Guinea, tariffs were compiled from third-party sources. For the Democratic Republic of the Congo, Eritrea, Somalia, Sudan, and the Republic of the Congo, where residential tariff structures were unavailable, average tariff values were adopted. All tariff data and sources are provided in the Electricity_Tariff_Calculator.xlsx dataset on Zenodo.

### Electricity bill estimation

Electricity tariff structures differ across countries. Most use social/lifeline tariffs and standard tariffs with either linear or increasing-block pricing for households, based on metrics such as electricity usage (kWh), active power (kW), apparent power (kVA), duration (hours), and current (A). Namibia (NAM) is the only country with clearly defined pensioner tariffs separate from its social/lifeline tariffs, shown on Supplementary Table 1.

Because residential demand data are often unreliable or even unavailable across most of SSA^42,43^, we use the MTF framework to estimate potential electricity bills for each energy tier based on prevailing residential tariff structures. The MTF provides comprehensive qualitative descriptions of energy service consumption across tiers^20,44^, but it does not specify numerical values for all metrics. Tier-specific current and apparent power can be derived from the defined electricity consumption and active power for each tier based on the MTF framework^20^ (see Equations (1), (2), and Table 1). For T1 to T4, active power is explicitly defined. For T5, we assume households utilize high-power appliances, with peak demand occurring when multiple appliances operate at the same time. Based on our calculations in Supplementary Table 5 and supporting literature^45^, we estimate a peak demand of approximately 14 kW before applying diversity adjustments.

**Table 1:** Electricity consumption and power requirements across MTF energy tiers^20,44^

	Tier 1	Tier 2	Tier 3	Tier 4	Tier 5
Load level	Very low load	Low load	Medium load	High load	Very high load
Daily electricity consumption (kWh)	Min 0.012	Min 0.2	Min 1	Min 3.4	Min 8.2
Monthly electricity consumption – 30 days (kWh)	Min 0.36	Min 6	Min 30	Min 102	Min 246
Active power (kW)	0.003–0.05	0.05–0.2	0.2–0.8	0.8–2	2–14
Apparent power (kVA)	0.003–0.053	0.053–0.211	0.211–0.842	0.842–2.105	2.105–14.737
Current (A)	0–0.139	0.139–0.554	0.554–2.226	2.226–5.540	5.540–38.781

	Tier 1	Tier 2	Tier 3	Tier 4	Tier 5
Duration per day (h)	Min 4	Min 4	Min 8	Min 16	Min 23
Possible service	Lighting, Phone charge, and Radio	Electrical lighting, Air circulation, Television, and Phone charging	Air cooler, Refrigerator, Food Processor, Water Pump, and Rice cooker	Washing machine, Iron, Hair dryer, Toaster, and Microwave	Air conditioner, Space heater, Vacuum cleaner, Water heater, and Electric Cookstove
Technology	Solar lantern	Stand-alone/Solar Systems	Generator/Mini-grid	Generator/Grid	Grid

The apparent power is derived using the power triangle formula:

1 $$S=\frac{P}{{{{\rm{PF}}}}}$$

where *S* denotes apparent power, *P* denotes active power, and PF denotes the power factor. A residential power factor of 0.95 is assumed^46^.

The current range for each tier is determined by the country-specific rated residential voltage^47^ and the MTF-defined active power, calculated based on Watt’s law:

2 $$I=\frac{P}{V\times {{{\rm{PF}}}}}$$

where *I* denotes current and *V* is voltage.

Electricity bills are estimated using one of two approaches, depending on the tariff structure:

- Consumption-based approach. For tariff structures based solely on electricity consumption (kWh), electricity bills are calculated using the minimum consumption threshold required for each MTF tier.
- Capacity-based approach. For tariff structures that include capacity restrictions—defined by active power (kW), apparent power (kVA), duration (hours), or current (A) to differentiate residential customer categories—monthly charges associated with maximum capacity levels (A, kVA, or kW) are incorporated alongside minimum electricity consumption requirements (kWh).

Therefore, electricity bills for different MTF tiers are calculated using the lowest applicable tariff at each consumption level, following the two approaches described above. When multiple tariff schedules exist, the average tariff is applied. Detailed calculation procedures for each country and tier are provided in the Electricity_Tariff_Calculator.xlsx dataset on Zenodo.

The electricity cost estimation is based on the following assumptions:

- Connection type. Only single-phase residential connections are considered, as they represent the standard configuration in most countries, while three-phase connections are primarily used in commercial and industrial settings. Although some countries (e.g., ZAF^48,49^) offer three-phase domestic tariffs, residential adoption remains limited.
- Tier scope. We focus primarily on grid electricity bills associated with T4 access, while T3 and T5 are treated as lower- and upper-bound sensitivity cases, respectively. This approach is better suited to assessing the potential for grid expansion and providing long-term guidance for achieving SDG7. By contrast, for T1 and T2 access levels, alternative off-grid solutions (e.g., solar PV systems and diesel generators) are generally more practical and cost-effective.
- Climatic influence. Climatic conditions may affect electricity consumption patterns; however, no data are currently available for this purpose.
- Currency and year standardization. All electricity tariff structures, originally reported in different currencies and reference years, are converted into 2024 US dollars using Equations (3) and (4).
  3 $${C}_{{y}_{0},{{{\rm{USD}}}}}=\frac{{C}_{{y}_{0},{{{\rm{Local}}}}}}{{{{{\rm{FX}}}}}_{{{{\rm{c}}}}}}$$
  4 $${C}_{i,{{{\rm{USD}}}}}={C}_{{y}_{0},{{{\rm{USD}}}}}\times {\prod }_{j={y}_{0}+1}^{i}\left(1+{\pi }_{j}^{{{{\rm{USD}}}}}\right)$$
  where ${C}_{{y}_{0},{{{\rm{USD}}}}}$ denotes the electricity bill in the base year *y*_0_, expressed in US dollar. The term ${\pi }_{j}^{{{{\rm{USD}}}}}$ represents the annual USD inflation rate in year *j*, where *j* runs from *y*_0_ + 1 to the target year *i* = 2024. The resulting value *C*_*i*,USD_ represents the inflation-adjusted electricity bill in the target year.

### Income distribution modelling

We model income distributions using log-normal distributions^50,51^, because it captures the positive skew and long right tail typical of income data, and also can be parametrized with limited inputs, as specified in Equation (5):

5 $$f(x,\mu,\sigma )=\frac{1}{x\sqrt{2\pi }\sigma }{e}^{-\frac{1}{2}{\left(\frac{\ln (x)-\mu }{\sigma }\right)}^{2}},\quad 0 < x < \infty$$

where *x* denotes monthly income per capita, while *μ* and *σ* denote the mean and standard deviation of the log-transformed income distribution, respectively. This model uses the Gini index, population size, and Gross National Income (GNI) per capita to account for economic disparities within country.

We use the Gini coefficient and monthly GNI data (Equation (6)) to calibrate the monthly income distribution and achieve a target income distribution. First, a set of random samples from the income log-normal distribution $y=\left\{{y}_{1},{y}_{2},\ldots,{y}_{n}\right\}$ is sorted in ascending order. Then, we use Equations (7)–(10) to calculate Gini coefficient, defined as *G*_current_, based on the initial monthly income distribution (IMID). In Equation (8), both relative income and population mapping are scaled to the range [0, 1]. Through iterative adjustments, the *G*_current_ gradually converge to *G*_target_. Finally, Equations (11) and (12) are used for model calibration.

6 $$M=\frac{{{{{\rm{GNI}}}}}_{{{{\rm{pc}}}},2024}}{12}$$

where *M* denotes monthly income per capita and GNI_pc,2024_ represents annual gross national income per capita in 2024.

7 $$L(k)=\frac{{\sum }_{f=1}^{k}{y}_{f}}{\mathop{\sum }_{f=1}^{n}{y}_{f}}$$

where *L*(*k*) denotes the cumulative income share; {*y*_1_, *y*_2_, …, *y*_*n*_} denotes the sorted income sequence, *n* denotes the population size, and *k* represents the cumulative population rank, where *k* = 1, …, *n*.

8 $$P(k)=\frac{k}{n}$$

where *P*(*k*) denotes the cumulative population share.

9 $$A=\int_{0}^{1}L(P)\,dP$$

where *A* denotes the area under the Lorenz curve.

10 $${G}_{{{{\rm{current}}}}}=1-2A$$

where *G*_current_ denotes the current Gini coefficient^52,53^.

11 $${{{\rm{AF}}}}={\left(\frac{{G}_{{{{\rm{target}}}}}}{{G}_{{{{\rm{current}}}}}}\right)}^{0.5}$$

where AF denotes the adjustment factor, *G*_target_ denotes the target Gini coefficient obtained from the World Bank (see ‘Data availability’), and *G*_current_ denotes the Gini coefficient calculated from the IMID model.

12 $${{{\rm{ID}}}}={x}^{{{{\rm{AF}}}}}\times \left(\frac{M}{\bar{x}}\right)$$

where ID denotes the adjusted monthly income distribution, *x* denotes the random variable generated within the IMID framework, and $\bar{x}$ represents the mean of the IMID distribution.

### Affordability calculation and assessment

Using the previously modelled income distribution, we calculate electricity affordability for each income percentile within the country. We adopt the widely recognized “Ten Percent Rule” as our affordability threshold, under which households spending 10% or more of the income on electricity are classified as energy poverty^24,25^. This criterion has been applied in various international studies^14,15,26–28^. To test robustness, we also conduct sensitivity analyses with 5% and 15% thresholds^54,55^, confirming that while absolute magnitudes shift, the geographic patterns and policy conclusions remain consistent.

The national average affordability is calculated as the mean share of electricity expenditure relative to income across all population percentiles, as defined in Equation (13):

13 $${A}_{t,c}=\frac{1}{100}\mathop{\sum }_{q=1}^{100}\left(\frac{{C}_{t}}{{I}_{c,q}}\right)$$

where *A*_*t*,*c*_ denotes the average share of electricity expenditure relative to income for country *c* at tier *t*, *C*_*t*_ denotes the monthly electricity cost for tier *t*, *q* denotes the population percentile (1–100), and *I*_*c*,*q*_ denotes the income associated with percentile *q* in country *c*.

All affordability calculations and income distribution modelling were implemented in Python using custom scripts archived on GitHub.

### Residential electricity demand

Residential electricity demand data are presented in Supplementary Table 4. For countries with available total residential electricity consumption data, per-household electricity demand was estimated by dividing total residential consumption by the number of households. Where only per capita electricity consumption data were available, total residential consumption was estimated by multiplying per capita consumption by the national population before deriving per-household demand using household numbers.

### Reporting summary

Further information on research design is available in the Nature Portfolio Reporting Summary linked to this article.

## Supplementary information

Supplementary Information

Reporting Summary

Transparent Peer Review file

## Data Availability

All data used in this study were obtained from publicly available sources without access restrictions. Net profit margin data were derived from UPBEAT^5^ using the average of the latest three years available for each country’s power utility. Only vertically integrated utilities (VIUs) and distribution utilities were included. Tariff data were collected from official utility websites or, where unavailable, from third-party sources, and are provided in the Electricity_Tariff_Calculator.xlsx on Zenodo. Data on exchange rates, Gini coefficients, GNI per capita, household size, inflation, population, and residential electricity consumption are provided in Supplementary Table 6. All data used in this study are available in the Zenodo database at 10.5281/zenodo.20122603.

## Code availability

The code for the simulated income distributions and tier-specific electricity affordability calculations in this study has been deposited in GitHub at 10.5281/zenodo.20348086^56^.
