# Supplementary material for: Assessing grid affordability for universal electricity access in Sub-Saharan Africa
Source: Nat Commun. 2026 Jul 9;17:8455. doi: 10.1038/s41467-026-75104-7 (PMC13478522; doi:10.1038/s41467-026-75104-7)
Supplement: Supplementary file 1 [file 41467_2026_75104_MOESM1_ESM.pdf]

Supplementary Information for

Assessing Grid Affordability for Universal Electricity Access in Sub-Saharan Africa

Yidan Kou[1], Stephanie Hirmer[1,*], Pu Yang[2], Alycia Leonard[1], Florian Egli[3,4*]

[1]Department of Engineering Science, University of Oxford,
Parks Road, Oxford OX1 3PJ, UK

[2]Institute of Sustainable Resources, The Bartlett School of Environment, Energy and Resources,
University College London, Central House, 14 Upper Woburn Place, London WC1H 0NN, UK

[3]School of Social Sciences and Technology,
Technical University of Munich, Richard-Wagner-Straße 1, 80333 Munich, Germany

[4]School of Management,
Technical University of Munich, Arcisstraße 21, 80333 Munich, Germany

*Corresponding authors: *stephanie.hirmer@eng.ox.ac.uk*
florian.egli@tum.de

Contents

Supplementary Figures

Supplementary Fig. 1: T3 Monthly residential electricity bills across SSA countries in 2024 USD ($). 2
Supplementary Fig. 2: T5 Monthly residential electricity bills across SSA countries in 2024 USD ($). 3
Supplementary Fig. 3: Utility financial health and T3 residential electricity affordability comparison. 4
Supplementary Fig. 4: Utility financial health and T4 residential electricity affordability comparison, including outliers. 5
Supplementary Fig. 5: Utility financial health and T5 residential electricity affordability comparison. 6

Supplementary Tables

Supplementary Table 1: Summary of residential electricity tariff structures across SSA 7
Supplementary Table 2: Population Share Able to Afford T4 and T5 Electricity under 5%, 10%, and 15% Thresholds. . 9
Supplementary Table 3: Average Electricity Affordability by Country and Energy Tiers. 10
Supplementary Table 4: Monthly Residential Electricity Demand and Energy Tiers by Country. 11
Supplementary Table 5: Maximum Possible Peak Demand for T5 Household Appliances. 12
Supplementary Table 6: Data sources for country indicators. 13

Supplementary References 14

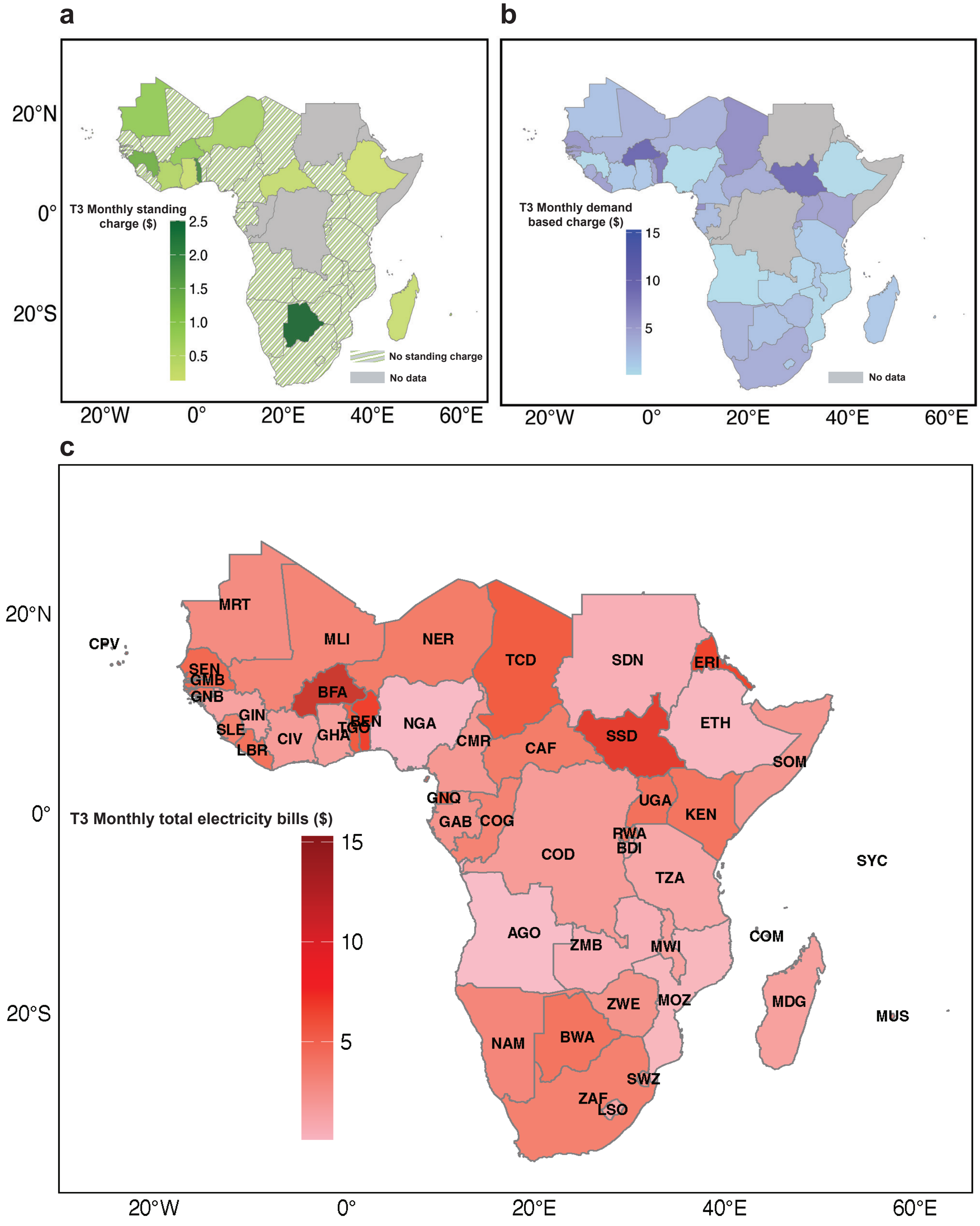

Supplementary Fig. 1. T3 Monthly residential electricity bills across SSA countries in 2024 USD ($). a, Monthly standing charges (fixed monthly fees paid by households). Green hatched areas indicate countries without standing charges. **b**, Monthly demand-based charges based on electricity consumption (kWh), active power (kW), apparent power (kVA) and current (A), including applicable taxes. **c**, Total electricity bills derived from panels **a** and **b**. For countries with missing data in panels **a** and **b** (grey areas), total bills in panel **c** were estimated using average residential electricity bills. In Liberia (LBR), Mali (MLI) and South Africa (ZAF), no standing charges apply to residential electricity consumption below 30 kWh per month owing to social/lifeline tariffs. For Guinea (GIN), postpaid tariffs with standing charges are shown. In Namibia (NAM), standard tariffs without standing charges are included, whereas the CENORED postpaid residential tariff (approximately US$8/month) is excluded. All underlying data are available on Zenodo in Summary_Electricity_Bills.xlsx. African administrative boundaries were obtained from Esri ArcGIS[1]. Countries are labelled using ISO alpha-3 codes.

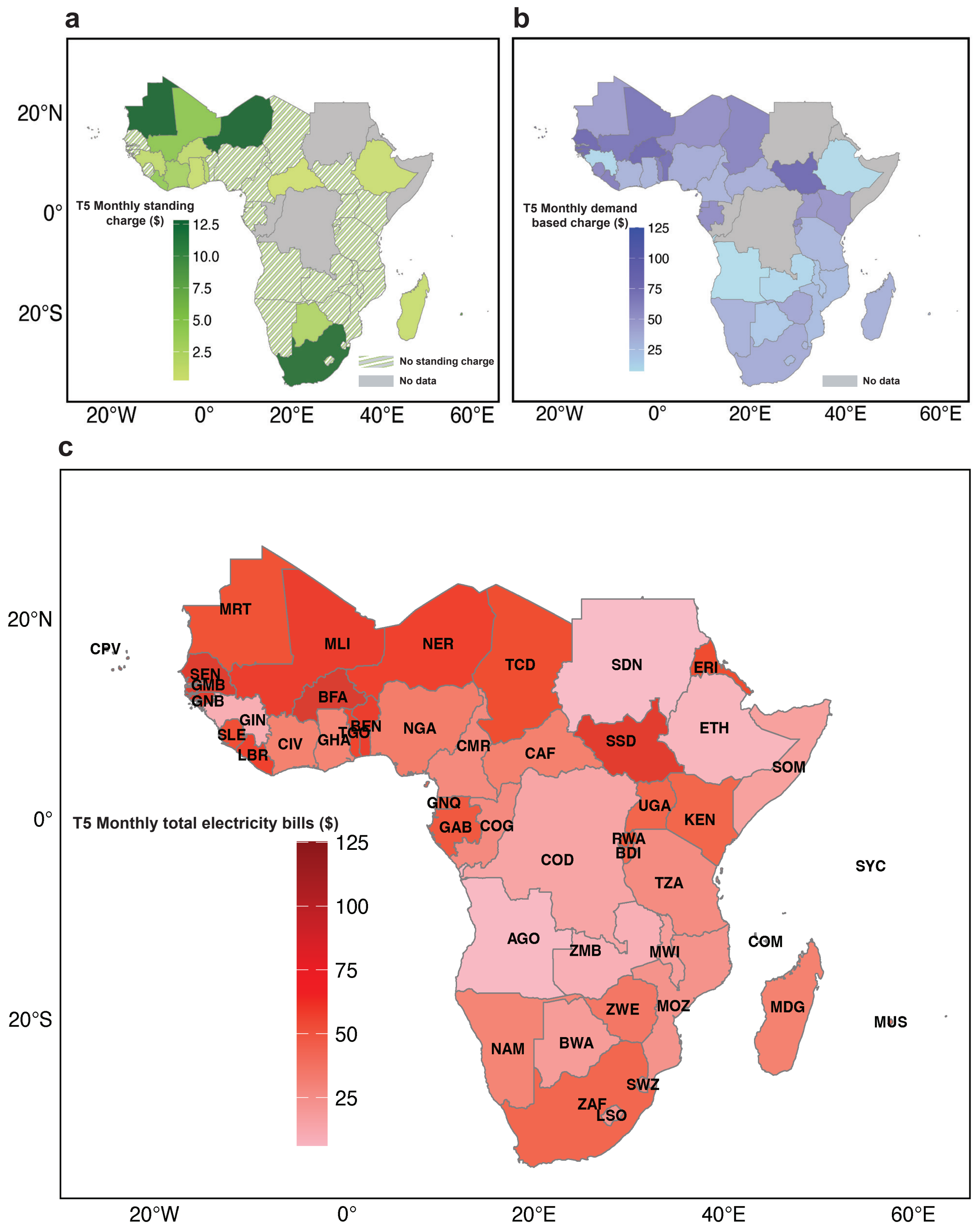

Supplementary Fig. 2. T5 Monthly residential electricity bills across SSA countries in 2024 USD ($). a, Monthly standing charges (fixed monthly fees paid by households). Green hatched areas indicate countries without standing charges. **b**, Monthly demand-based charges based on electricity consumption (kWh), active power (kW), apparent power (kVA) and current (A), including applicable taxes. **c**, Total electricity bills derived from panels **a** and **b**. For countries with missing data in panels **a** and **b** (grey areas), total bills in panel **c** were estimated using average residential electricity bills. For Guinea (GIN) and Mali (MLI), postpaid and prepaid tariffs with standing charges are shown, respectively. In Namibia (NAM), standard tariffs without standing charges are included, whereas the CENORED postpaid residential tariff (approximately US$8/month) is excluded. All underlying data are available on Zenodo in Summary_Electricity_Bills.xlsx. African administrative boundaries were obtained from Esri ArcGIS[1]. Countries are labelled using ISO alpha-3 codes.

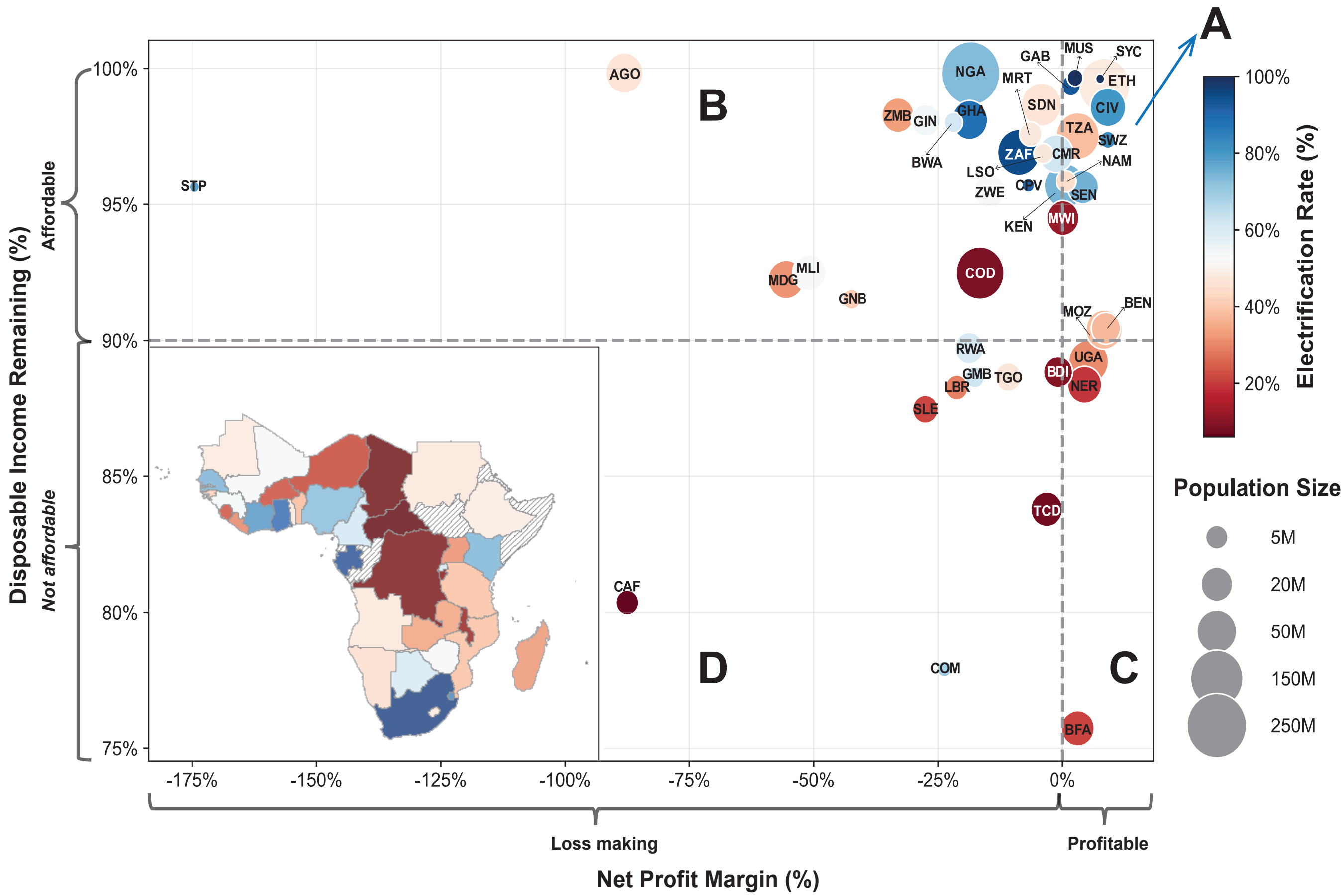

Supplementary Fig. 3. Utility financial health and T3 residential electricity affordability comparison. The x-axis shows the net profit margin of distribution utilities or vertically integrated utilities (VIUs), used as an indicator of utility financial health, with data obtained from UPBEAT[2]. The y-axis shows the percentage of household income remaining after T3 electricity expenditure (30 kWh/month). The dashed horizontal line at 90% represents the affordability threshold, indicating that electricity expenditure does not exceed 10% of household income. And the dashed vertical line at zero net profit margin represents the financial health threshold. Within this framework, the four quadrants illustrate different electricity access conditions and challenges: the upper-right quadrant (**A**) represents grid electrification opportunity; the upper-left quadrant (**B**) indicates utility stress; the lower-right quadrant (**C**) reflects affordability stress; and the lower-left quadrant (**D**) represents a grid electrification trap. Bubble size represents population and colour indicates national electrification rate. Net profit margin data were unavailable for Equatorial Guinea (GNQ), Eritrea (ERI), Republic of the Congo (COG), Somalia (SOM), and South Sudan (SSD), which are indicated by grey parallel lines in the inset African map. African administrative boundaries were obtained from Esri ArcGIS[1]. Countries are labelled using ISO alpha-3 codes.

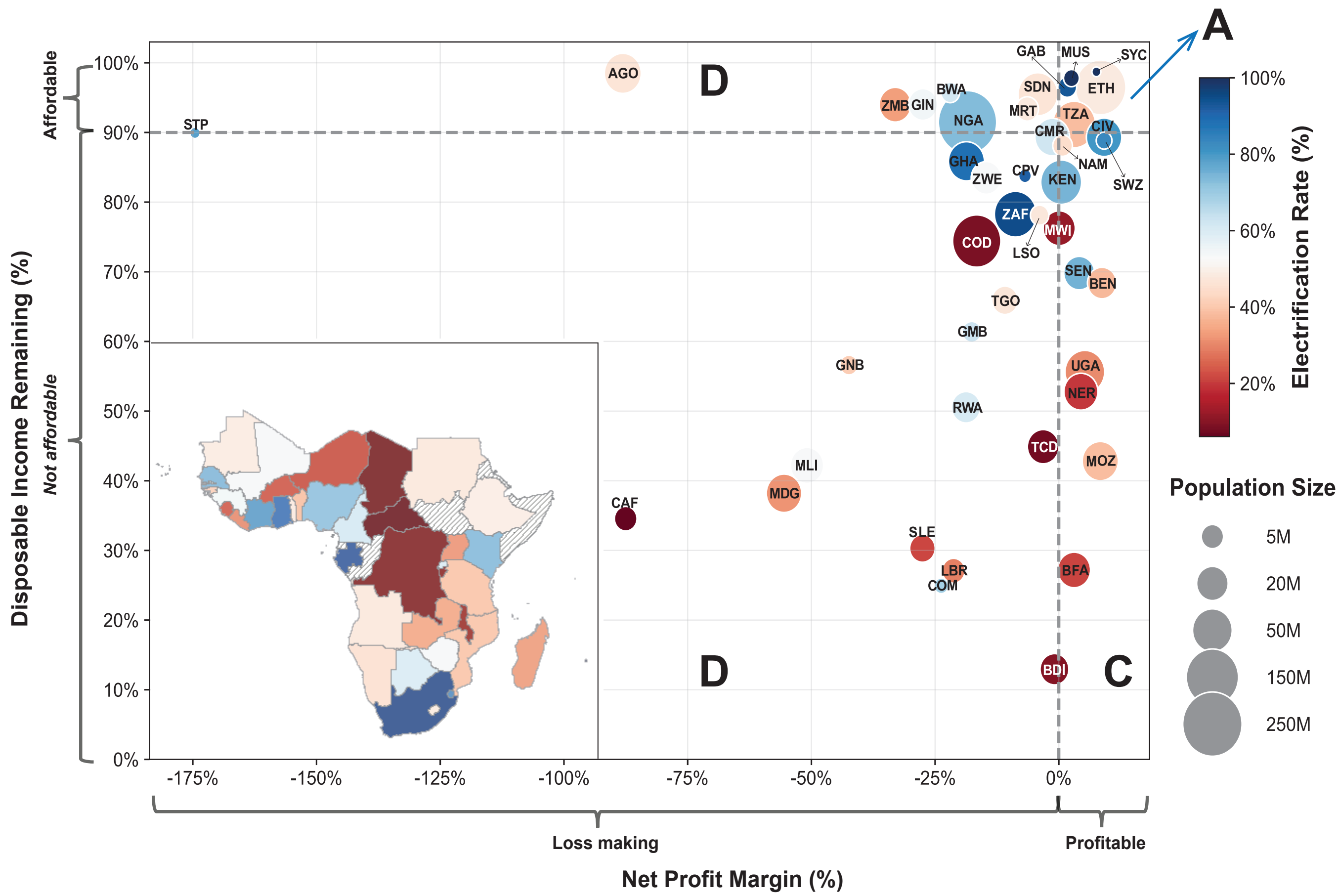

Supplementary Fig. 4. Utility financial health and T4 residential electricity affordability comparison, including outliers. The x-axis shows the net profit margin of distribution utilities or vertically integrated utilities (VIUs), used as an indicator of utility financial health, with data obtained from UPBEAT[2]. The y-axis shows the percentage of household income remaining after T3 electricity expenditure (102 kWh/month). The dashed horizontal line at 90% represents the affordability threshold, indicating that electricity expenditure does not exceed 10% of household income. And the dashed vertical line at zero net profit margin represents the financial health threshold. Within this framework, the four quadrants illustrate different electricity access conditions and challenges: the upper-right quadrant (**A**) represents grid electrification opportunity; the upper-left quadrant (**B**) indicates utility stress; the lower-right quadrant (**C**) reflects affordability stress; and the lower-left quadrant (**D**) represents a grid electrification trap. Bubble size represents population and colour indicates national electrification rate. Net profit margin data were unavailable for Equatorial Guinea (GNQ), Eritrea (ERI), Republic of the Congo (COG), Somalia (SOM), and South Sudan (SSD), which are indicated by grey parallel lines in the inset African map. African administrative boundaries were obtained from Esri ArcGIS[1]. Countries are labelled using ISO alpha-3 codes.

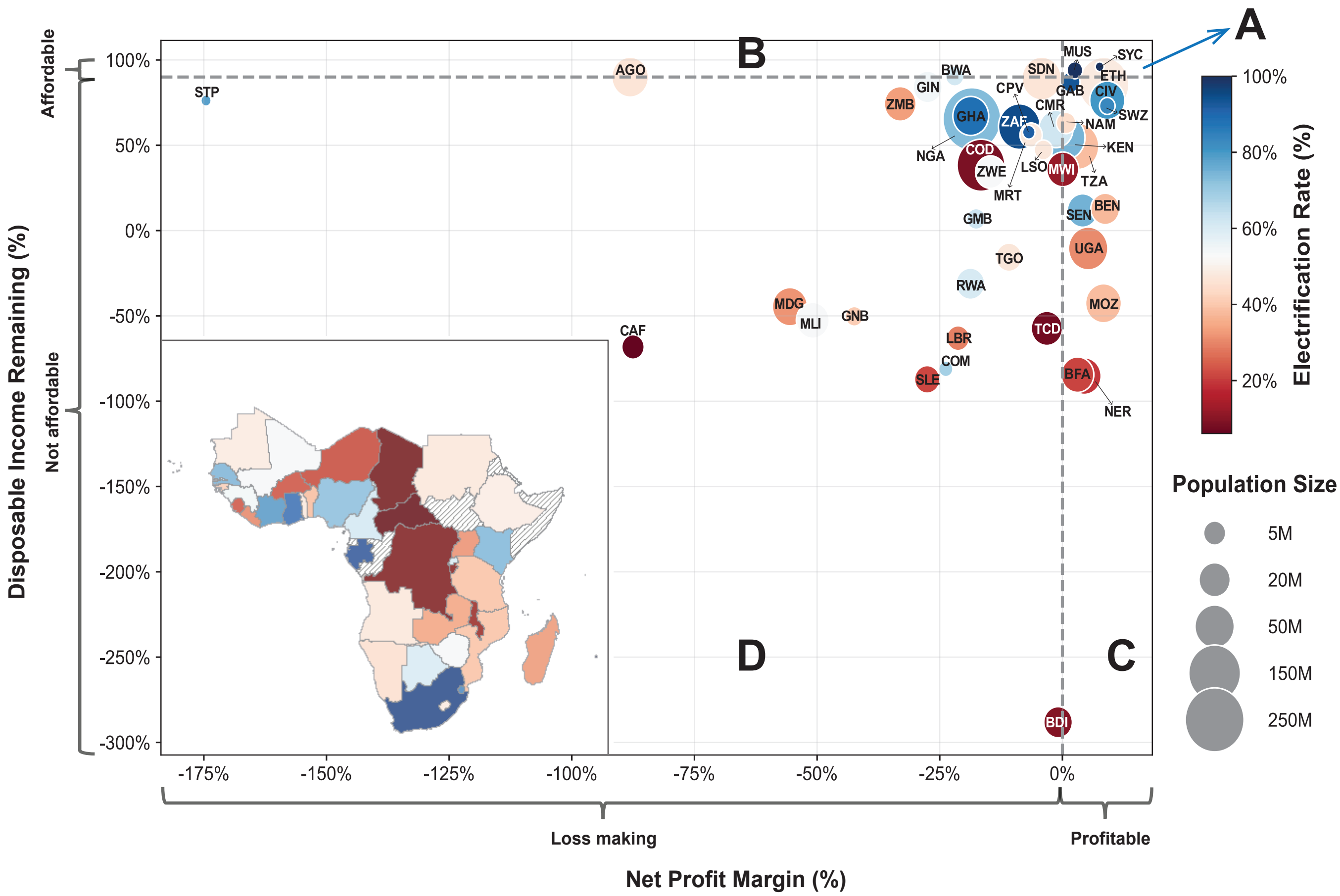

Supplementary Fig. 5. Utility financial health and T5 residential electricity affordability comparison The x-axis shows the net profit margin of distribution utilities or vertically integrated utilities (VIUs), used as an indicator of utility financial health, with data obtained from UPBEAT[2]. The y-axis shows the percentage of household income remaining after T3 electricity expenditure (246 kWh/month). The dashed horizontal line at 90% represents the affordability threshold, indicating that electricity expenditure does not exceed 10% of household income. And the dashed vertical line at zero net profit margin represents the financial health threshold. Within this framework, the four quadrants illustrate different electricity access conditions and challenges: the upper-right quadrant (**A**) represents grid electrification opportunity; the upper-left quadrant (**B**) indicates utility stress; the lower-right quadrant (**C**) reflects affordability stress; and the lower-left quadrant (**D**) represents a grid electrification trap. Bubble size represents population and colour indicates national electrification rate. Net profit margin data were unavailable for Equatorial Guinea (GNQ), Eritrea (ERI), Republic of the Congo (COG), Somalia (SOM), and South Sudan (SSD), which are indicated by grey parallel lines in the inset African map. African administrative boundaries were obtained from Esri ArcGIS[1]. Countries are labelled using ISO alpha-3 codes.

Supplementary Table 1. Summary of residential electricity tariff structures across SSA

Country	Social tariff (Yes/No)	Standing charge (Yes/No)	Tariff structure	No. of tariff schedules	Capacity-related criteria for block assignment (Yes/No)	Capacity indicator used for block assignment	Capacity-based charging (LCU/kVA LCU/kW LCU/Amp)
AGO	No	No	IBT (4 blocks)	4	Yes	KVA	Kz/KVA
BDI	No	No	IBT (3 blocks)	1	No	——	——
BEN	Yes	No	Linear	3	Yes	KVA	FCFA/kVA
BFA	Yes	Yes	Linear & IBT (3 blocks)	5	Yes	Amp	CFA/Amp
BWA	No	No	IBT (2 blocks)	1	No	——	——
CAF	No	Yes	IBT (3 blocks)	1	No	——	——
CMR	No	No	IBT (4 blocks)	1	No	——	——
COG	Avg. residential tariff applied						
COD	Avg. residential tariff applied						
COM	No	No	Linear	1	No	——	——
CPV	No	No	IBT (2 blocks)	1	No	——	——
CIV	Yes	Yes	Linear	8	Yes	kVA	——
ERI	Avg. residential tariff applied						
ETH	No	Yes	prepaid & postpaid IBT (7 blocks)	2	No	——	——
GAB	Yes	No	Linear	6	No	——	——
GHA	Yes	Yes	IBT (3 blocks)	2	No	——	——
GIN	No	Yes	prepaid IBT (2 blocks) & postpaid IBT (3 blocks)	2	Yes	kVA	——
GNB	No	No	IBT (3 blocks)	1	No	——	——
GMB	No	No	IBT (4 blocks)	1	No	——	——
GNQ	No	No	Linear		No	——	——
KEN	Yes	No	IBT (3 blocks)	3	No	——	——
LSO	Yes	No	Linear	2	No	——	——
LBR	Yes	Yes	prepaid & postpaid Linear	3	No	——	——
MDG	Yes	Yes	IBT (2 blocks)	9	Yes	kW	——
MLI	No	Yes	IBT (2 blocks & 4 blocks)	2	Yes	Amp & kVA	——
MOZ	Yes	No	prepaid Linear & postpaid IBT (3 blcoks)	4	No	——	——
MRT	Yes	Yes	Linear	8	Yes	kVA	——

Continued on next page

Supplementary Table 1 – continued from previous page

Country	Social or lifeline tariff (Yes/No)	Standing charge (Yes/No)	Tariff structure	No. of tariff schedules	Capacity-related criteria for block assignment (Yes/No)	Capacity indicator used for block assignment	Capacity-based charging (LCU/kVA LCU/kW LCU/Amp)
MWI	No	Yes	prepaid & postpaid IBT (2 blocks)	2	No	——	——
MUS	Yes	Yes	IBT (12 blocks) - total 4 tariff schedules applied	4	Yes	kW	——
NAM	Yes	Yes	Linear & IBT (3 blocks)	12 (3 utilities)	Yes	Amp	N$/Amp/month
NER	Yes	Yes	IBT (3 blocks)	6	Yes	kW	——
NGA	Yes	No	Linear	5	No	——	——
RWA	No	No	IBT (3 blocks)	1	No	——	——
SEN	No	No	IBT (3 blocks)	5	Yes	kW	FCFA/kW
SLE	Yes	No	IBT (3 blocks)	1	No	——	——
SOM	Avg. residential tariff applied						
SSD	No	No	IBT (2 blocks)	1	No	——	——
SDN	Avg. residential tariff applied						
STP	No	Yes	prepaid Linear & postpaid IBT (3 blcoks)	2	Yes	A	——
SYC	No	No	IBT (5 blocks)	3	Yes	kVA	Sr/ kVA
SWZ	Yes	No	IBT (3 blocks)	2	No	——	——
TCD	No	No	IBT (2 blocks)	1	No	——	——
TGO	Yes	Yes	IBT (4 blocks)	3	Yes	kVA	FCFA/kVA/month
TZA	No	No	IBT (2 blocks)	1	No	——	——
UGA	No	No	IBT (4 blocks)	1	No	——	——
ZAF	No	No	IBT (2 blocks)	12	Yes	Network capacity charge	R/POD/day
ZMB	No	No	IBT (4 blocks)	1	No	——	——
ZWE	No	No	IBT (6 blocks)	1	No	——	——

Social/lifeline tariff indicates preferential electricity tariffs for low-income or low-usage households. Standing charge refers to a fixed monthly fee. Tariff structure describes either a linear tariff (single unit rate) or an increasing block tariff (IBT), where the unit price changes with electricity consumption level. No. of tariff schedules shows the number of residential tariff categories. Capacity-related criteria for block assignment indicates whether customer classification is based on kVA, kW, or Ampere ratings. Capacity-based charging shows whether charges depend on contracted capacity. All data and related sources are available on Zenodo in Electricity_Tariff_Calculator.xlsx:. Countries are labelled using ISO alpha-3 codes.

Supplementary Table 2. Population Share Able to Afford T4 and T5 Electricity under 5%, 10%, and 15% Thresholds

Country	Tier 4 (5%)	Tier 4 (10%)	Tier 4 (15%)	Tier 5 (5%)	Tier 5 (10%)	Tier 5 (15%)
AGO	96	100	100	39	67	80
BDI	1	1	2	1	1	1
BEN	1	7	19	1	1	1
BFA	1	1	3	1	1	1
BWA	73	90	96	44	70	82
CAF	1	3	7	1	1	1
CPV	13	40	60	1	1	17
CMR	27	61	79	2	8	18
COD	6	23	40	1	4	10
COG	25	52	69	6	19	33
COM	1	3	7	1	1	1
CIV	19	58	79	2	15	34
ERI	1	1	3	1	1	1
ETH	80	98	100	9	39	64
GAB	80	97	100	15	48	70
GHA	18	48	67	3	14	28
GIN	46	89	98	3	27	56
GMB	1	6	16	1	1	2
GNB	1	2	7	<1	1	1
GNQ	34	62	77	9	27	42
KEN	8	33	56	1	4	11
LBR	1	1	2	1	1	1
LSO	9	29	47	1	6	14
MDG	1	3	8	1	1	1
MLI	1	1	4	1	1	1
MOZ	2	9	17	1	1	3
MRT	41	84	96	1	2	6
MUS	94	100	100	51	86	95
MWI	4	19	37	1	2	5
NAM	41	65	77	12	28	40
NER	1	1	5	1	1	1
NGA	30	71	88	1	5	16
RWA	1	6	14	1	1	2
SDN	67	94	99	17	56	79
SEN	1	9	23	1	1	1
SLE	1	1	2	<1	1	1
SOM	2	14	32	1	1	4
SSD	1	5	13	1	1	2
STP	28	63	81	5	21	39
SWZ	39	65	78	14	32	47
SYC	100	100	100	75	97	100
TCD	1	2	6	1	1	1
TGO	1	8	20	1	1	1
TZA	34	69	85	1	4	10
UGA	1	7	16	1	1	2
ZAF	28	48	61	15	31	42
ZMB	60	83	92	12	30	46
ZWE	21	46	63	2	7	14

Affordability thresholds represent electricity spending equal to 5%, 10%, and 15% of income, respectively. Population shares indicate the proportion of the population able to afford grid-connected electricity at T4 and T5 under each threshold, based on the income distribution modelling and affordability assessment (see Methods). Countries are labelled using ISO alpha-3 code.

Supplementary Table 3. Average Electricity Affordability by Country and Energy Tiers.

Country	Tier 1 (%)	Tier 2 (%)	Tier 3 (%)	Tier 4 (%)	Tier 5 (%)
AGO	0.002054	0.034229	0.171145	1.516229	10.052614
BDI	0.133988	2.233131	11.165657	87.055816	388.147296
BEN	0.112580	1.229276	9.554171	31.616168	87.313670
BFA	4.848470	6.687084	24.265137	72.806171	184.446504
BWA	1.051949	1.230307	1.989276	4.266185	9.358037
CAF	1.505359	4.955724	19.638127	65.444436	168.204048
CIV	0.365220	0.567772	1.429693	10.696774	23.654905
CMR	0.037621	0.627014	3.135068	10.659230	40.486201
COD	0.090309	1.505142	7.525710	25.587412	61.710818
COG	0.049287	0.821451	4.107257	13.964673	33.679505
COM	0.265000	4.416665	22.083326	75.083310	181.083276
CPV	0.051601	0.860012	4.300059	16.221632	42.352536
ERI	0.242611	4.043518	20.217589	68.739801	165.784226
ETH	0.210369	0.292117	0.639982	3.527983	14.445024
GAB	0.007619	0.126989	0.634945	3.488548	12.998147
GHA	0.200834	0.523800	1.898125	14.137900	32.815493
GIN	0.783276	0.997696	1.910121	5.983403	15.810443
GMB	0.136347	2.272449	11.362245	38.631632	93.170408
GNB	0.101869	1.697819	8.489097	43.395703	150.260503
GNQ	0.039804	0.663404	3.317018	11.277860	27.199544
KEN	0.051789	0.863143	4.315715	17.135444	45.550716
LBR	0.140849	2.347490	11.737449	72.915642	163.059252
LSO	0.037574	0.626237	3.131186	21.874735	52.756714
MDG	0.876923	1.593815	7.761285	61.795945	144.620808
MLI	0.090134	1.502236	7.511178	57.791007	152.630333
MOZ	0.115258	1.920970	9.604848	57.180284	142.612875
MRT	0.685709	1.015044	2.416466	6.620734	44.037049
MUS	0.109451	0.151790	0.346762	2.202298	6.026731
MWI	0.066029	1.100490	5.502448	23.748506	64.117686
NAM	0.816552	1.456001	4.150083	11.895184	36.969853
NER	1.552868	3.471326	11.634980	47.213588	185.282827
NGA	0.002179	0.036309	0.181546	8.530979	34.672145
RWA	0.073007	1.216779	10.287939	49.452743	131.154699
SDN	0.015936	0.265608	1.328038	4.515329	10.889910
SEN	0.057688	0.961459	5.865040	30.192927	88.239515
SLE	0.123290	2.054837	12.532250	69.706406	187.200953
SOM	0.085784	1.429731	7.148655	24.305427	58.618971
SSD	0.187091	3.118191	15.590953	53.075023	132.648087
STP	2.025102	2.469533	4.360726	10.062116	23.803928
SWZ	0.031543	0.525721	2.628607	11.171760	26.943656
SYC	0.004569	0.076157	0.380787	1.294675	3.950710
TCD	0.194496	3.241601	16.208003	55.107209	157.312968
TGO	3.623195	5.100193	11.350098	34.094769	115.633906
TZA	0.029815	0.496913	2.484564	8.861611	50.602285
UGA	0.061834	1.030561	10.783785	44.369066	110.483234
ZAF	0.037053	0.617552	3.087761	21.734309	39.061859
ZMB	0.020660	0.344339	1.721693	6.012882	25.734091
ZWE	0.053949	0.899157	4.495787	16.522057	65.713416

Affordability is calculated as the share of electricity bills relative to household income for each population income percentile (see Methods). National averages for each energy tier are rounded half up to six decimal places. Yellow rows indicate countries where the national average affordability for T4 or T5 electricity is no more than 10% of household income. Countries are labelled using ISO alpha-3 code.

Supplementary Table 4. Monthly Residential Electricity Demand and Energy Tiers by Country.

Country	Monthly Residential Demand (kWh)	Energy Tier
AGO	128	Tier 4
BDI	16	Tier 2
BEN	6	Tier 1
BFA	10	Tier 2
BWA	255	Tier 5
CAF	13	Tier 2
CIV	57	Tier 3
CMR	36	Tier 3
COD	6	Tier 1
COG	50	Tier 3
COM	19	Tier 2
CPV	261	Tier 5
ERI	28	Tier 2
ETH	21	Tier 2
GAB	145	Tier 4
GHA	58	Tier 3
GIN	29	Tier 2
GMB	94	Tier 3
GNB	10	Tier 2
GNQ	260	Tier 5
KEN	19	Tier 2
LBR	17	Tier 2
LSO	84	Tier 3
MDG	8	Tier 2
MLI	101	Tier 3
MOZ	19	Tier 2
MRT	145	Tier 4
MUS	348	Tier 5
MWI	17	Tier 2
NAM	174	Tier 4
NER	19	Tier 2
NGA	24	Tier 2
RWA	4	Tier 1
SDN	84	Tier 3
SEN	132	Tier 4
SLE	35	Tier 3
SOM	14	Tier 2
SSD	6	Tier 1
STP	102	Tier 4
SWZ	390	Tier 5
SYC	1046	Tier 5
TCD	2	Tier 1
TGO	17	Tier 2
TZA	18	Tier 2
UGA	9	Tier 2
ZAF	314	Tier 5
ZMB	114	Tier 4
ZWE	38	Tier 3

Estimated monthly residential electricity demand and corresponding MTF energy tiers by country. Monthly demand values are rounded to the nearest integer for tier classification. Countries are labelled using ISO alpha-3 code.

Supplementary Table 5. Maximum Possible Peak Demand for T5 Household Appliances.

Household Appliance	Min Power (W)	Max Power (W)
Window air conditioner[3]	900	1440
Refrigerator[4]	300	800
Electric cookstove[5]	1000	3000
Vacuum cleaner[6]	600	2000
LED lighting[7]	10	10
Washing machine[8]	400	1400
Microwave[9]	600	1000
Oven[10]	2000	5000
Electric water heating[11]	2000	4000
TV[12]	50	200
Laptop[13]	30	70
**Sum**	7890	18920

All listed appliances are assumed to be used at the same time under T5 consumption conditions, and the maximum possible peak demand is calculated before applying diversity.

Supplementary Table 6. Data sources for country indicators.

Indicator	Country	Source
Exchange rate	AGO, BEN, BFA, BDI, BWA, CAF, CIV, CMR, COD, COG, COM, CPV, ERI, ETH, GAB, GHA, GMB, GNB, GNQ, KEN, LBR, LSO, MDG, MLI, MOZ, MUS, NAM, NER, RWA, SDN, SEN, SOM, SSD, STP, SWZ, SYC, TCD, TGO, UGA, ZAF, ZMB	World Bank[14]
	GIN, MWI, NGA, SLE, TZA	ExchangeRates[15]
	MRT	World Food Programme[16]
	ZWE	Trading Economics[17]
Gini coefficient	AGO, BEN, BFA, BDI, BWA, CAF, CIV, CMR, COD, COG, COM, CPV, ETH, GAB, GHA, GIN, GMB, GNB, KEN, LBR, LSO, MDG, MLI, MOZ, MRT, MUS, MWI, NAM, NGA, NER, RWA, SDN, SEN, SLE, SSD, STP, SWZ, SYC, TCD, TGO, TZA, UGA, ZAF, ZMB, ZWE	World Bank[18]
	SOM	CEIC[19]
	ERI	Confidus Solutions[20]
	GNQ	Statista[21]
GNI per capita	AGO, BEN, BFA, BDI, BWA, CAF, CIV, CMR, COD, COG, COM, CPV, ETH, GAB, GHA, GIN, GMB, GNB, GNQ, KEN, LBR, LSO, MDG, MLI, MOZ, MRT, MUS, MWI, NAM, NGA, NER, RWA, SDN, SEN, SLE, SOM, SSD, STP, SWZ, SYC, TCD, TGO, TZA, UGA, ZAF, ZMB, ZWE	World Bank[22]
	ERI	Trading Economics[23]
Household size	AGO, BEN, BFA, BDI, BWA, CAF, CIV, CMR, COD, COG, COM, CPV, ERI, ETH, GAB, GHA, GIN, GMB, GNB, GNQ, KEN, LBR, LSO, MDG, MLI, MOZ, MRT, MUS, MWI, NAM, NGA, NER, RWA, SDN, SEN, SLE, SOM, SSD, STP, SWZ, TCD, TGO, TZA, UGA, ZAF, ZMB, ZWE	Global Data Lab[24]
	SYC	National Bureau of Statistics[?]
Inflation rate	USA	US Inflation Calculator[25]
Population	All 48 SSA countries	World Bank[26]
Residential electricity demand	AGO, BEN, BFA, BWA, CIV, CMR, COD, COG, ERI, ETH, GAB, GHA, KEN, MDG, MOZ, MUS, NAM, NER, NGA, RWA, SDN, SEN, SSD, TCD, TGO, TZA, UGA, ZAF, ZMB, ZWE	IEA[27]
	BDI, CAF, COM, CPV, GIN, GMB, GNB, GNQ, LBR, LSO, MLI, MRT, MWI, SLE, SOM, STP, SWZ, SYC	Index Mundi[28]

Data sources for exchange rates, Gini coefficients, GNI per capita, household size, inflation rates, population, and residential electricity demand. Countries are referenced using ISO alpha-3 codes.

Supplementary References

1. ESRI. World countries generalized. Available at https://hub.arcgis.com/datasets/esri::world-countries-generalized (2026). Accessed: May. 15, 2026.
2. UPBEAT. Utility Performance and Behavior Today. *World Bank Group*. Available at https://utilityperformance.energydata.info (2024). Accessed: May. 15, 2026.
3. TCL. How much electricity does an air conditioner consume? Available at https://www.tcl.com/uk/en/blog/how-much-electricity-does-an-air-conditioner-use (2023). Accessed: May. 15, 2026.
4. Marsh, J. How many watts does a refrigerator use? *Energysage*. Available at https://www.energysage.com/electricity/house-watts/how-many-watts-does-a-refrigerator-use (2026). Accessed: May. 15, 2026.
5. Renogy. How many watts does an electric stove use? Available at https://www.renogy.com/blog/how-many-watts-does-an-electric-stove-use (2024). Accessed: May. 15, 2026.
6. May, T. How many watts of power is good for a vacuum cleaner? *Techradar*. Available at https://www.techradar.com/home/vacuums/how-many-watts-of-power-is-good-for-a-vacuum-cleaner (2024). Accessed: May. 15, 2026.
7. Marsh, J. How many watts does a light bulb use? *Energysage*. Available at https://www.energysage.com/electricity/house-watts/how-many-watts-does-a-light-bulb-use (2024). Accessed: May. 15, 2026.
8. Marsh, J. How many watts does a washing machine use? *Energysage*. Available at https://www.energysage.com/electricity/house-watts/how-many-watts-does-a-washing-machine-use (2024). Accessed: May. 15, 2026.
9. Marsh, J. How many watts does a microwave use? *Energysage*. Available at https://www.energysage.com/electricity/house-watts/how-many-watts-does-microwave-use (2024). Accessed: May. 15, 2026.
10. Marsh, J. How many watts does an electric oven use? *Energysage*. Available at https://www.energysage.com/electricity/house-watts/how-many-watts-does-an-electric-oven-and-stove-use (2024). Accessed: May. 15, 2026.
11. Jackery. How many watts does a water heater use? https://www.jackery.com/blogs/knowledge/how-many-watts-does-a-water-heater-use (2026). Accessed: May. 15, 2026.
12. Marsh, J. How many watts does a tv use? *Energysage*. Available at https://www.energysage.com/electricity/house-watts/how-many-watts-does-a-tv-use (2024). Accessed: May. 15, 2026.
13. Marsh, J. How many watts does a computer use? *Energysage*. Available at https://www.energysage.com/electricity/house-watts/how-many-watts-does-a-computer-use (2024). Accessed: May. 15, 2026.
14. World Bank. Official exchange rate (LCU per US$, period average). Available at https://data.worldbank.org/indicator/PA.NUS.FCRF (2024). Accessed: May. 15, 2026.
15. Exchange Rates. Foreign currency exchange rates. Available at https://www.exchangerates.org.uk. Accessed: May. 15, 2026.
16. World Food Programme. Mauritania - exchange rates. Available at https://dataviz.vam.wfp.org/western-africa/mauritania/economic/exchange-rates (2024). Accessed: May. 15, 2026.
17. Trading Economics. ZIGUSD Zimbabwe Gold US Dollar. Available at https://tradingeconomics.com/zigusd:cur (2024). Accessed: May. 15, 2026.
18. World Bank. Poverty & Inequality Indicators. Available at https://pip.worldbank.org/poverty-calculator (2022). Accessed: May. 15, 2026.
19. CEIC. Somalia Gini Coefficient. Available at https://www.ceicdata.com/en/somalia/social-gini-coefficient-annual/gini-coefficient (2023). Accessed: May. 15, 2026.
20. Confidus Solution. Economy of Eritrea. Available at https://www.confiduss.com/en/jurisdictions/eritrea/economy/ (2026). Accessed: May. 15, 2026.
21. Statista. Socioeconomic Indicators - Equatorial Guinea | Forecast. Available at https://www.statista.com/outlook/co/socioeconomic-indicators/equatorial-guinea. Accessed: May. 15, 2026.
22. World Bank. Country profile. Available at https://pip.worldbank.org/country-profiles (2024). Accessed: May. 15, 2026.
23. Trading Economics. Eritrea - GNI per capita, Atlas Method. Available at https://tradingeconomics.com/eritrea/gni-per-capita-atlas-method-us-dollar-wb-data.html (2024). Accessed: May. 15, 2026.

24. Global Data Lab. Household Size Data - Selected African Countries. Available at https://globaldatalab.org/areadata/table/hhsize/AGO+BEN+BWA+BFA+BDI+CPV+CMR+CAF+TCD+COM+COD+COG+CIV+GNQ+ERI+SWZ+ETH+GAB+GMB+GHA+GIN+GNB+KEN+LSO+LBR+MDG+MWI+MLI+MRT+MUS+MOZ+NAM+NER+NGA+RWA+STP+SEN+SLE+SOM+ZAF+SSD+SDN+TZA+TGO+UGA+ZMB+ZWE/?levels=1 (2024). Accessed: May. 15, 2026.

25. US Inflation Calculator. Current US Inflation Rates: 2000-2025. Available at https://www.usinflationcalculator.com/inflation/current-inflation-rates. Accessed: May. 15, 2026.

26. World Population Review. List of Countries in Africa - 2024 Ranking. Available at https://worldpopulationreview.com/country-rankings/countries-in-africa (2026). Accessed: May. 15, 2026.

27. International Energy Agency. Africa energy insights. Available at https://www.iea.org/regions/africa (2026). Accessed: May. 15, 2026.

28. Index Mundi. Electricity consumption per capita - global rankings. Available at https://www.indexmundi.com/g/r.aspx?v=81000 (2024). Accessed: May. 15, 2026.
