# Supplementary material for: Assessing grid affordability for universal electricity access in Sub-Saharan Africa
Source: Nat Commun. 2026 Jul 9;17:8455. doi: 10.1038/s41467-026-75104-7 (PMC13478522; doi:10.1038/s41467-026-75104-7)
Supplement: Supplementary file 3 [file 41467_2026_75104_MOESM3_ESM.pdf]

nature portfolio

Peer Review File

Assessing Grid Affordability for Universal Electricity Access in Sub-Saharan Africa

Corresponding Author: Professor Stephanie Hirmer

This file contains all reviewer reports in order by version, followed by all author rebuttals in order by version.

Version 0:

Reviewer comments:

Reviewer #1

(Remarks to the Author)
This is a very well-executed analysis of why observed electricity usage in Sub-Saharan Africa (SSA) is so low. The authors consider both the affordability of electricity, as determined by utility rate structures, relative to income, and the difficulty of sustaining and increasing service provision when utilities are not financially viable given rate structures and observed levels of consumption. Either or both factors are shown to be important for many SSA countries, though some countries appear to be in a good position to more pro-actively expand the power grid to increase electricity use. Other countries, burdened with both affordability and utility viability challenges, could be well-advised to pursue well-targeted off-grid alternatives.
The paper employs a solid methodology and significantly extends prior research on the topic. It will be useful for both researchers and policy advisors seeking to address the underlying challenges to accelerating SSA economic development through increased electricity access and use.
I have a few technical questions related to the methods used in the paper, which would be useful to address in the paper but may not involve any revision. I also have a few questions related to the analytical and policy conclusions.
Methods
Pages 15-16: Are there any significant sources of potential bias in using the lognormal distribution to model income distributions? For example, are some lower household incomes under-stated because household consumption includes self-provided goods? Does tax evasion lead to any downward bias in modeling higher incomes?
Page 16: I am not sure why there is a need to construct an SSA-wide income distribution model. Cannot results for the whole region on affordability not be inferred from country-level distributions?
Page 17: The 10% rule for energy poverty is commonly used, but it is still ad hoc. How do the results change if a different poverty threshold is used – would there be any surprises?
Page 17: Why would electricity use increase linearly in family size, as implied by the transformation of per capita to household figures? For example, electricity for cooling a home with a fan would not increase proportionately in comparing a household of 2 versus 3. If I have understood correctly, it seems like there are figures for total household electricity consumption in numerous countries (page 18), which could be converted to average per-household by dividing by the number of households for comparison.
Conclusions
Page 7: “The differences in affordability between countries arise primarily because of varying household income, rather than electricity bills.” Does this mean, then, that despite the significant differences in tariff structures across groups I--IV, there are few or no cases where countries have similar income distributions but electricity is much more affordable in one than the other because of tariff differences? If so, that is surprising and warrants discussion.
Page 9: “Specifically, in Ethiopia, Guinea, and Tanzania—where households can theoretically afford T4 electricity—average monthly household electricity demand remains at 21 kWh, 29 kWh, and 18 kWh, respectively, which corresponds to Tier 2 usage.” Page 10: “This combination of consumer affordability and utility financial sustainability presents a favourable opportunity for Gabon to pursue last-mile electrification through grid expansion.”
Both comments reflect an important missing ingredient in the paper – the differences between rural and urban electrification. In Ethiopia, the lack of rural grid extension – not just a last-mile problem – is a major barrier to increased electricity use. Rural grid extension is expensive, even for utilities that are well-run. Without distinguishing between rural and urban circumstances for countries with significant under-served rural populations, it is difficult to say the issue is just affordability based on tariff designs. The paper could do more to develop this distinction.
Also with respect to the observation about Gabon – to what extent does something similar hold for other countries in Quadrant A?

Page 11: "In such contexts, off-grid solutions, particularly through public–private partnerships, may offer a more viable path to electrification." I know that the paper is focused more on the affordability and utility viability analysis than on documenting solutions, but – what sorts of partnerships? Why? These statements need some explanation.
Page 12: "Low demand may stem from household energy preferences (e.g., charcoal for cooking in rural areas), or limited purchasing power to acquire the appliances necessary for higher tier electricity usage." Household preferences may play some role, but appliance affordability is critical. In this case, electric cooking typically is expensive – even in urban areas.
Page 12: "Tariff structures that fail to reflect geographic and socioeconomic heterogeneity can unintentionally worsen these inequities. Therefore, electrification planning must explicitly incorporate local context and variability." I think this is actually a more complicated issue. What is "geographic heterogeneity" aside from socio-economic heterogeneity? If electricity is costlier in one region than another, why cannot a NATIONAL utility arbitrage those differences in a national power market? It may be because the utility is not actually national in its coverage and inter-connectedness. Does it then follow that the utility has geographically heterogeneous markets, or should it invest in becoming fully national?
Page 12: "Extending services into low-affordability areas without complementary demand stimulation—such as subsidies for connection fees or appliance financing schemes—risks further fiscal strain for utilities, creating a feedback loop of underutilisation and declining investment." This statement would be clearer if the authors referred back to the conclusions reached by comparing tariff structures. Having high energy charges – well above variable costs – is inefficient and a major disincentive for demand expansion. However, high energy charges often are an effort to offset low standing charges put in place to stimulate connection. These in turn undermine utility financial viability, while high energy charges dissuade households from connecting. A better rate design is energy charges in line with variable cost and higher standing charges, potentially accompanied by TARGETED connection subsidies for lower-income households – and along with this, attention to cost-effective non-grid solutions.
Page 13: "Public–private partnerships, particularly in off-grid contexts, can help deliver sustainable outcomes without overburdening national utilities." Again, this somewhat vague statement needs better explanation.

Reviewer #2

(Remarks to the Author)
This manuscript presents a macro-level evaluation of grid electricity affordability in Sub-Saharan Africa (SSA), a critical factor for achieving universal electricity access (SDG 7.1). The study compiles and analyzes an averaged dataset of household electricity tariffs across 48 SSA countries, categorizing them by household Multi-Tier Framework (MTF) demand levels. It juxtaposes affordability with approximations of usage and utility financial health, aiming to highlight challenges and opportunities for electricity access through grid extension. The paper is well-written and easy to read. While it offers significant insights, the current methodology and scope may not fully meet the high-quality standards typically required for a Nature Communications publication.

Noteworthy Strengths and Results:
•Tariff Dataset: The paper boasts the development of what it claims to be the most comprehensive electricity tariff database for SSA to date, covering both standing and demand-based charges. This dataset is a valuable contribution, even if primarily based on the UPBEAT World Bank database.
•Widespread Affordability Gap: A important finding is that under current national grid tariffs, 51% of SSA's total population cannot afford Tier 4 electricity by grid connection, and this rises to 86% for Tier 5. This clearly highlights a substantial barrier to achieving higher-tier electrification even for the under-grid population.
•Income as Primary Driver of Affordability: The study effectively demonstrates that differences in affordability between countries are primarily driven by varying household incomes rather than electricity bills themselves. This is a crucial insight for policy formulation.
•Discrepancy Between Affordability and Demand: The analysis reveals that theoretical affordability does not always translate into actual demand. Even in countries where high-tier electricity is theoretically affordable, actual usage often falls short, underscoring significant supply-side challenges such as grid reliability, high connection fees, and the upfront costs of appliances.
•Utility Health and Affordability Quadrants: The introduction of a framework categorizing countries into four quadrants (grid electrification opportunity, utility stress, affordability stress, and grid electrification trap) is highly valuable offering actionable insights for targeted policy interventions.

Significance to the Field and Comparison to Established Literature

This work is highly significant to the fields of energy access, development economics, and sustainable development. It builds upon established literature concerning electricity access in SSA (e.g., Blimpo et al., 2020; Winkler et al., 2011). Some additional and updated references, such as Afrobarometer 9april24-1.pdf instead of the 2022 Afrobarometer, would provide a stronger background. It is also important to include established literature on affordability for electricity in SSA, for instance, "Mapping of affordability levels for photovoltaic-based electricity generation in the solar belt of sub-Saharan Africa, East Asia and South Asia" (Nature-Scientific Reports, 2021).

A few times, grey literature is used instead of peer-reviewed papers. A few examples:
•The cited situation of under-grid connection in Kenya from K Lee et al., "Electrification for "Under Grid" households in Rural Kenya, Development Engineering, Volume 1, 2016, 26-35" states: "For example, in Kenya, a study showed that although the electricity grid covered 80% of households in the surveyed areas, only 50% had a physical connection".
•On line 45, a news on a master's thesis on gender is cited to justify the assumption that "T4 is assumed to be the lowest demand level that would justify grid expansion as opposed to off-grid electrification". It is highly recommended to strengthen

this assumption with more robust existing literature.
•On Line 30, the statement "Even worse, arbitrary or unsubstantiated tariff-setting can drive high-consuming customers off-grid (e.g., as seen by South Africa's Eskom)" refers to the "positive" move in South Africa to PV caused by increasing instability of the grid. The growth of solar is a key reason why there has been less load-shedding in 2024.

Weaknesses and Areas for Improvement:
•Lack of Granular Contextualization: While the paper provides interesting findings at a country level, it lacks country-specific contextualization.
•Role of Decentralized Energy and Subsidies: The paper does not adequately discuss the role of decentralized energy solutions in addressing the affordability gap, nor the impact of subsidies on government budgets versus utilities (private/national). While briefly mentioned in the discussion, these critical aspects are not integrated into the methods or supplementary information.
•Affordability Calculation Granularity: The affordability calculations, particularly the "Ten Percent Rule," use national average data for income expenditure rather than specific income tiers for households. This overlooks potential differences between rural/urban populations or those close/far from the grid. As highlighted, lower-income households consistently spend a significantly higher percentage of their income on energy, and a national average might obscure the true extent of affordability challenges for vulnerable groups.
•Peak Demand Assumptions: The assumptions for Peak Demand for Energy appear to be very rough, using a basic basket of appliances without sufficiently accounting for wealth divergences and climatic conditions between countries. This could affect the accuracy of the estimated electricity usage tiers.

Version 1:

Reviewer comments:

Reviewer #1

(Remarks to the Author)
I appreciate the efforts of the authors in responding to my comments on the previous version of the paper. I have no additional suggestions.

(Remarks on code availability)

Reviewer #2

(Remarks to the Author)
The paper presents a valuable compilation of country-level data, and this is certainly one of its strengths. However, the analytical component remains relatively limited. The conclusions on affordability rely primarily on national averages, although energy poverty varies substantially across rural and urban contexts. Similarly, the affordability calculations use national tariff levels and TIER consumption tiers, which may not fully capture the considerable heterogeneity in consumption patterns across countries and subnational regions. As a result, the analysis does not fully reflect the complexity of affordability dynamics or their spatial disparities.

As an example, Figure 2 provides a useful high-level overview of tariff variations by grouping countries into four categories; however, the distinctions among social, domestic, pensioner, and general tariffs are not easily interpretable in the current format. The discussion could be strengthened by incorporating the influence of energy policy and regulatory frameworks—such as different subsidy schemes—on affordability outcomes. At present, the findings mainly confirm broad regional patterns across TIER levels, with affordability being closely aligned with the poverty proxy used.

In summary, while the datasets are of clear value and the topic is important, the manuscript may be better suited to a specialized journal where data-driven analyses of energy access are a central focus for the readership. I hope the authors find this feedback constructive and helpful in refining their work further for another Journal.

(Remarks on code availability)

Version 2:

Reviewer comments:

Reviewer #2

(Remarks to the Author)
Having reviewed the manuscript through two rounds, my opinion is that it is a solid piece of work but lacks the degree of conceptual novelty or groundbreaking insight necessary for Nature Communications. The authors have assembled compelling and useful datasets, which could serve as a resource for other researchers and academics. I leave the final editorial decision to you regarding the best path forward.

(Remarks on code availability)

Open Access This Peer Review File is licensed under a Creative Commons Attribution 4.0 International License, which permits use, sharing, adaptation, distribution and reproduction in any medium or format, as long as you give appropriate credit to the original author(s) and the source, provide a link to the Creative Commons license, and indicate if changes were made.
In cases where reviewers are anonymous, credit should be given to 'Anonymous Referee' and the source.
The images or other third party material in this Peer Review File are included in the article's Creative Commons license, unless indicated otherwise in a credit line to the material. If material is not included in the article's Creative Commons license and your intended use is not permitted by statutory regulation or exceeds the permitted use, you will need to obtain permission directly from the copyright holder.
To view a copy of this license, visit https://creativecommons.org/licenses/by/4.0/

Response to reviewers: Manuscript NCOMMS-25-31084A

We would really like to thank the reviewers for taking the time to provide such detailed and constructive comments. Many valuable points were raised and we feel that this review has greatly improved the manuscript. A detailed response to each reviewer comment is provided below. Original comments are shown in black, responses are provided in blue, and revisions to the manuscript are marked in red.

Response to Reviewer 1

Reviewer Comment 1.1 — Pages 15-16: Are there any significant sources of potential bias in using the lognormal distribution to model income distributions? For example, are some lower household incomes under-stated because household consumption includes self-provided goods? Does tax evasion lead to any downward bias in modeling higher incomes?

Reply: Thank you for this thoughtful question. We acknowledge that any modelling approach to income distributions introduces potential sources of bias, particularly in contexts where informal economic activity and non-monetary household production are significant. This may lead to underestimation of lower incomes when using monetary measures alone. Similarly, informality and tax evasion can contribute to downward bias in reported higher incomes.

Our choice of the lognormal distribution is grounded in its wide acceptance in the literature, where empirical studies using actual data have shown that 97–99% of the income distribution (low–middle incomes) fits a lognormal model across three macro-datasets [Clementi and Gallegati, 2005]. We acknowledge that the upper tail (highest incomes) may deviate [Eguia and Xefteris, 2024]; however, this has limited impact on our analysis, as our focus is on the lowest income groups that struggle most with electricity affordability. In contexts where standardized microdata are unavailable across all countries, the lognormal distribution provides a tractable and consistent way to capture the typical skewness of income distributions, using systematically available inputs that reflect the situation of the majority of the population in Sub-Saharan Africa (SSA). While we recognize that alternative approaches can sometimes capture inequality more precisely (e.g., Generalized Beta distributions), these require microlevel datasets that are not consistently available.

To clarify this, we have added a brief explanation in the Methods section, acknowledging these potential biases and justifying why the lognormal remains the most appropriate, transparent, and replicable choice under current data constraints.

- Page 20, line 341-347:

  "Our methodology is designed to provide a transparent and comparable framework for assessing electricity affordability in 48 SSA countries, despite limited data availability. We rely on country-specific tariff structures and model income distributions using widely applied methods (e.g., lognormal) to ensure consistency. Although the modeled distribution inevitably simplifies some within-country heterogeneity, it allows us to capture broad regional patterns and test affordability across tiers in a way that is both replicable and robust."

- Page 23-24, line 402-404:

  "We model income distributions using a lognormal distribution [50,51], because it captures the positive skew and long right tail typical of income data, and also can be parameterized with limited inputs, as specified in Formula 5."

$$f(x, \mu, \sigma) = \frac{1}{x\sqrt{2\pi}\sigma} e^{-\frac{1}{2}\left(\frac{\ln(x)-\mu}{\sigma}\right)^2}, \quad 0 < x < \infty \tag{5}$$

Reviewer Comment 1.2 — Page 16: I am not sure why there is a need to construct an SSA-wide income distribution model. Cannot results for the whole region on affordability not be inferred from country-level distributions?

Reply: Thanks for raising this point. The SSA-wide income distribution in our study is not intended to serve as an independent analytical model. Its sole purpose is to provide an intuitive, visual representation of the overall proportion of people in the region who can and cannot afford electricity under the given tariff assumptions. The curve is simply an aggregation of all country-level income distributions. All affordability calculations and analyses are based on country-level distributions. As we are unable to include curves for all countries in the main text, this curve alongside the example countries provided offers a useful illustration. We have clarified this in the Figure 3 and Methods as follows:

- Figure 3 Caption, Page 12:

  "The SSA-wide income distribution is simply the aggregation of all country-level distributions for illustrative purposes only."

- Methods, Page 25, line 430-433:

  "To provide an intuitive, regional-level illustration, we also construct an aggregated SSA income distribution. This is not used in affordability calculations, which are conducted at the country level, but is included to visually summarize the overall affordability challenge across the region."

Reviewer Comment 1.3 — Page 17: The 10% rule for energy poverty is commonly used, but it is still ad hoc. How do the results change if a different poverty threshold is used – would there be any surprises?

Reply: Thanks for this question. In response, we would first like to justify the use of the 10% rule, and then explain what happens when different thresholds are used.

First, while the 10% rule may be perceived to be arbitrary and normative, it is widely adopted as a global standard. It therefore remains a useful reference point for defining electricity affordability. As referenced in the draft, this threshold is used in numerous international studies, e.g., Bednar and Reames [2020], Galvin [2024], L.Taylor [1993], Muhihi [2024], Simshauser [2021], Winkler et al. [2011], Zhang et al. [2019].

Second, to test the robustness of this threshold, we conducted a sensitivity analysis using 5% and 15% as thresholds for the proportion of household income spent on energy to induce energy poverty, as shown in the Supplementary Table S1. These thresholds capture a reasonable tolerance range. In Tier 4, the share of households unable to afford electricity in SSA ranges from 38%-71% across the 5%–15% affordability thresholds (i.e., 33% range), while in Tier 5 this range increases to 76%–97% (i.e., 19% range). While these shifts are substantial, the overall patterns and country rankings remain, and the key policy conclusions of the study are unchanged.

We therefore conclude that the 10% threshold is acceptable, and changing it does not lead to qualitative "surprises" in the geographic distribution of affordability stress, instead mainly altering the number of affected people. We has revised the abstract (page 1), 'Affordability' section (page 9 - 11) and Methods (page 25) as follows:

- Abstract, Page 1, line 1-18:

  "Grid expansion is seen as a key strategy for increasing electricity access across Sub-Saharan Africa (SSA). However, whether utilities can generate sufficient revenue from customers under current tariffs to support the capital investment for grid expansion is unclear. Assessing this conundrum, this study compiles and analyzes household electricity tariffs for the 48 countries in SSA categorized by household demand levels within the Multi-Tier Framework and household affordability thresholds set as 5%, 10%, and 15%. Under current tariffs, approximately 608 million people (50%) cannot afford Tier 4 (T4) electricity, rising to 1.05 billion (85%) for full Tier 5 (T5) access under 10% energy poverty threshold. Sensitivity checks at 5% and 15% indicate that while absolute numbers vary widely (38% to 71% for T4; 76% to 97% for T5), overall trends and relative country rankings remain consistent. Moreover, even where higher-tier electricity is affordable, usage remains low. In countries with both low affordability and weak utility, grid expansion appears unfeasible. While, in some instances, targeted policy reforms and tariff adjustments may provide a path forward and offer potential remedies to overcome these challenges, off-grid solutions may be the only viable option."

- Page 9, line 138-150:

  "To evaluate the affordability of grid electricity bills, we simulate income distributions for each country and estimate the share of the population that could afford T4 or T5 electricity access under 5%, 10%, and 15% thresholds (see Methods and Supplementary Table S1). In SSA, the share of population unable to afford T4 electricity is approximately 71% at the 5% threshold, 50% at the 10% threshold, and 38% at the 15% threshold. For T5 access, the corresponding unaffordability shares rise to 97%, 85%, and 76%, respectively. The size of the population deemed unable to afford electricity is highly sensitive to the affordability threshold applied, which in turn reflects the share of disposable income that households at different income levels are willing or able to allocate to electricity. Although absolute values differ, the overall patterns and relative rankings across countries remain consistent across all affordability thresholds. Since the 10% is widely adopted as the benchmark for defining energy poverty, we adopt this as the primary reference in the following analysis."

- Page 9-10, line 158-168:

  "Affordability is especially severe in countries such as Burkina Faso (BFA), Burundi (BDI), the Central African Republic (CAF), Chad (TCD), Comoros (COM), Eritrea (ERI), Guinea-Bissau (GNB), Liberia (LBR), Madagascar (MDG), Mali (MLI), Niger (NER), and Sierra Leone (SLE), where less than 10% of the population can afford electricity across all income thresholds. By contrast, countries such as Angola (AGO), Mauritius (MUS), Seychelles (SYC) demonstrate relatively higher affordability, with over 90% of the population theoretically able to cover the associated electricity costs. The affordability challenge becomes even more severe at the Tier 5 level, where the number of people unable to afford electricity rises to 1.05 billion—86% of the population—and most countries experience sharp declines across all income thresholds."

- Page 11, line 186-191:

  "Furthermore, the choice of affordability threshold strongly influences the estimated size of the unaffordable population, even though broader affordability challenges remain evident. Some countries, such as Guinea, Mauritania, and Nigeria, display relatively high affordability under the 10% and 15% thresholds but experience sharp declines when the stricter 5% threshold is applied."

- Methods, Page 25, line 443-449:

  "We adopt the widely recognized "Ten Percent Rule" as our affordability threshold, which suggests that spending 10% or more of a household's income on electricity indicates energy poverty [54,55]. This criterion has been applied in various international studies [14,15,56,57,58]. To test robustness, we also conduct sensitivity analyses with 5% and 15% thresholds [59,60], confirming that while absolute magnitudes shift, the geographic patterns and policy conclusions remain consistent."

Reviewer Comment 1.4 — Page 17: Why would electricity use increase linearly in family size, as implied by the transformation of per capita to household figures? For example, electricity for cooling a home with a fan would not increase proportionately in comparing a household of 2 versus 3. If I have understood correctly, it seems like there are figures for total household electricity consumption in numerous countries (page 18), which could be converted to average per-household by dividing by the number of households for comparison.

Reply: We acknowledge that this was not adequately explained in the previous version and agree that using actual household-level consumption data, where available, provides a more accurate basis. However, such data were not available. Instead, we made use of two different data types. In 30 countries where data on total residential electricity usage was available, this was directly divided by the number of households to estimate average per-household usage. In 18 countries where this was not available, we turned to residential electricity usage per capita, which we multiplied by the national population and then divided by the estimated number of households to estimate average per-household usage.

In the new draft, we have updated and refined our explanation for estimating per-household consumption to reflect that this limitation is based on data availability. We updated the Methods and the calculation in the "Demand" Excel available on Zenodo.

Page 26, line 457-464:

"For 30 countries, we obtained total residential electricity usage data from the International Energy Agency (IEA) [58] and estimated per-household usage by dividing this total by the number of households in each country. For the remaining 18 countries without IEA data, we use residential electricity usage per capita from the Index Mundi database [59]. We then derive total residential electricity usage by multiplying this per capita value by the national population, and subsequently divide by the estimated number of households to obtain per-household usage."

Reviewer Comment 1.5 — Page 7: "The differences in affordability between countries arise primarily because of varying household income, rather than electricity bills." Does this mean, then, that despite the significant differences in tariff structures across groups I–IV, there are few or no cases where countries have similar income distributions but electricity is much more affordable in one than the other because of tariff differences? If so, that is surprising and warrants discussion.

Reply: Thanks for this insightful comment. Our results indicate that differences in affordability across SSA countries are overwhelmingly driven by variations in household income rather than electricity tariffs. This is due to the different orders of magnitude involved: cross-country income differences often span several orders of magnitude, whereas tariff levels typically range only within one order of magnitude (i.e., between \$0.05–0.60/kWh). As a result, income effects dominate affordability outcomes.

In principle, tariff differences could matter if countries with similar income distributions had very different tariffs. However, we do not observe such cases: in examples like Rwanda and Uganda, both income distributions and tariffs are broadly comparable, leading to similar affordability outcomes. This reinforces the conclusion that income, rather than tariffs, is the predominant determinant of affordability across the region. We have added a clarification in the "Affordability" section as follows:

- Page 10, line 173-175:

  "Cross-country differences in affordability stem primarily from disparities in household income, while differences in electricity tariffs play a comparatively smaller role—even though, in principle, tariff structures could also influence affordability."

- Page 10-11, line 181-186:

  "Notably, we do not find evidence of countries with broadly similar income distributions but divergent affordability outcomes driven by tariffs alone. In cases such as Rwanda and Uganda, or Madagascar and Mozambique, both incomes and tariffs are broadly comparable, leading to convergent affordability outcomes. This reinforces the finding that income is the predominant determinant of affordability results."

Reviewer Comment 1.6 — Page 9: "Specifically, in Ethiopia, Guinea, and Tanzania—where households can theoretically afford T4 electricity—average monthly household electricity demand remains at 21 kWh, 29 kWh, and 18 kWh, respectively, which corresponds to Tier 2 usage."

Page 10: "This combination of consumer affordability and utility financial sustainability presents a favourable opportunity for Gabon to pursue last-mile electrification through grid expansion."

Both comments reflect an important missing ingredient in the paper – the differences between rural and urban electrification. In Ethiopia, the lack of rural grid extension – not just a last-mile problem – is a major barrier to increased electricity use. Rural grid extension is expensive, even for utilities that are well-run. Without distinguishing between rural and urban circumstances for countries with significant under-served rural populations, it is difficult to say the issue is just affordability based on tariff designs. The paper could do more to develop this distinction. Also with respect to the observation about Gabon – to what extent does something similar hold for other countries in Quadrant A?

Reply: We thank the reviewer for highlighting this important factor in assessing electrification strategies. The main reason we did not conduct a detailed rural–urban affordability analysis is the lack of consistent and comparable data across SSA. Specifically:

(1) reliable statistics on the share of rural grid-connected versus off-grid users are missing; and

(2) no standardized dataset exists for off-grid generation tariffs that could be directly compared with national grid tariffs.

For these reasons, our analysis relies on nationally aggregated grid-connected data as the only benchmark consistently available across countries.

However, we recognize the importance of discussing rural–urban differences. Therefore, we have revised the "Balancing Utility Health and Tariff Affordability" section for Quadrant **A** (pages 13–15) to incorporate these aspects. Specifically:

(1) We demonstrate the importance of geographical location and the cost of different electrification options when considering the viability of grid expansion under different national circumstances, and illustrate this through case studies.

(2) We have tightened the focus of the paper to emphasize that our modeling concerns grid electricity tariffs only, and we have clarified that our discussion adopts a macroscopic perspective, identifying contexts where grid expansion could be theoretically feasible based on affordability and utility health. Within this framework, countries in Quadrant **A** exhibit high potential for grid expansion, particularly when rural areas are located near existing grid infrastructure and the associated costs remain acceptable—both of which warrant further investigation. In Quadrants **B** and **C**, structural barriers already constrain grid expansion regardless of these differences. For Quadrant **D**, where both affordability and utility health are weak, further disaggregation is not meaningful, as utilities cannot operate sustainably in such contexts.

Future research could build on this work by examining selected countries with richer datasets to conduct a more detailed geographical rural–urban analysis.

Page 14-16, line 228-253:

"First, countries in the *grid electrification opportunity* quadrant (**A**) have approximately 110 million people who lack access to electricity. Expanding the grid could bring substantial benefits to underserved region. However, it is essential to carefully consider the geographic distribution of uneletrified housholds, even though our analysis indicates that countries in quadrant **A** show high potential for grid expansion. To illustrate, in Gabon (GAB), households spend on average only 3% of their income to afford T4 electricity, while the national utility, Société d'Énergie et d'Eau du Gabon (SEEG), maintains a profit margin of 1.7% relative to revenue. These results suggest that the utility may have sufficient financial capacity to invest in grid expansion, and that households are likely able to afford electricity at the current tariff level. However, by 2023, although Gabon had achieved a national electrification rate of 94% [24], rural areas lagged significantly behind, with only 29% coverage [25]. Rural residents are widely dispersed across tropical rainforests and hilly terrains, whereas power generation and transmission systems are concentrated in coastal and central cities such as Libreville, Port-Gentil, and Franceville. This spatial mismatch reduces the economic viability of grid expansion to remote rural areas. A significant rural–urban electrification disparity is also observed in Ethiopia (ETH) and Tanzania (TZA) [24,25,26]. These cases demonstrate that utilities' overall affordability and profitability outcomes are largely driven by urban households. To achieve universal energy access, greater attention should be given to rural areas, with careful consideration of distance and cost in electrification strategies—particularly in relation to the size of underserved regions and their proximity to the existing grid infrastructure. There is also a possibility that revenues from urban consumers may need to be leveraged to cross-subsidize rural electrification, a dimension that requires further analysis. By contrast, in smaller island states such as Mauritius (MUS) and Seychelles (SYC), grid expansion can more readily achieve last-mile connections."

Reviewer Comment 1.7 — Page 11: "In such contexts, off-grid solutions, particularly through public–private partnerships, may offer a more viable path to electrification." I know that the paper is focused more on the affordability and utility viability analysis than on documenting solutions, but – what sorts of partnerships? Why? These statements need some explanation.

Page 13: "Public–private partnerships, particularly in off-grid contexts, can help deliver sustainable outcomes without overburdening national utilities." Again, this somewhat vague statement needs better explanation.

Reply: We agree that our original statements on public–private partnerships (PPPs) to facilitate off-grid solutions on pages 11 and 13 were quite general. To clarify, we have now defined PPPs within the context of this paper on page 16, i.e., being an example of an approach. Furthermore, we have clarified that the primary focus of this paper is the viability of grid-based electrification, which may not have been clear in the previous version.

We have revised the abstract, literature review, and discussion sections to:

(1) underscore the importance of off-grid electrification as a distinct and viable option when assessing countries in SSA across all quadrants of Figure 5,

(2) describe the implementation mechanism through ownership–operation splits,

(3) provide real-world policy references, and

(4) highlight the broader conclusion that feasible electrification strategies must be aligned with local demand profiles and institutional realities in order to achieve meaningful progress toward electrification.

- Abstract, Page 1-2, line 1-18:

  Grid expansion is seen as a key strategy for increasing electricity access across Sub-Saharan Africa (SSA). However, whether utilities can generate sufficient revenue from customers under current tariffs to support the capital investment for grid expansion is unclear. Assessing this conundrum, this study compiles and analyzes household electricity tariffs for the 48 countries in SSA categorized by household demand levels within the Multi-Tier Framework and household affordability thresholds set as 5%, 10%, and 15%. Under current tariffs, approximately 608 million people (50%) cannot afford Tier 4 (T4) electricity, rising to 1.05 billion (85%) for full Tier 5 (T5) access under 10% energy poverty threshold. Sensitivity checks at 5% and 15% indicate that while absolute numbers vary widely (38% to 71% for T4; 76% to 97% for T5), overall trends and relative country rankings remain consistent. Moreover, even where higher-tier electricity is affordable, usage remains low. In countries with both low affordability and weak utility, grid expansion appears unfeasible. While, in some instances, targeted policy reforms and tariff adjustments may provide a path forward and offer potential remedies to overcome these challenges, off-grid solutions may be the only viable option."

- Page 3, line 48-58:

  "This is important, as the grid continues to constitute a viable and often indispensable option—not necessarily for last-mile distribution in sparsely populated or remote areas, but particularly in regions characterized by higher population density and greater consumption potential, where economies of scale can be more effectively realized. We focus specifically on grid-based electrification, as most countries continue to regard it as the default strategy to address low access rates, and it is our intention here to evaluate the validity of this approach. Nevertheless, this emphasis should not be interpreted as overlooking the potential and importance of off-grid solutions, which remain critical where grid expansion proves infeasible and may, in such contexts, represent the more effective pathway to universal access."

- Page 3-4, line 62-65:

  "Acknowledging that tariffs are likely to rise as connection rates increase—given the high capital cost of reaching harder-to-serve populations—we use current tariffs to present a maximally optimistic view of affordability."

- Page 17, line 276-281:

  "In such contexts, off-grid or mini-grid solutions, particularly through public–private partnerships, may offer a more viable path to electrification. By adopting a model in which the

government owns the system while private investors operate and maintain it, national governments can share both capital and operational responsibilities. This arrangement reduces the financial burden on public utilities while helping to ensure a more stable and reliable electricity supply."

- Page 19, line 331-339:

  "In many cases—though not the primary focus of this paper—off-grid solutions may prove more viable. Such solutions have already been successfully integrated in several SSA countries, including Tanzania, Mali, and Ghana [35,36]. The success of electrification strategies should be judged not by the number of connections but by the affordability and reliability of the energy services delivered. Aligning interventions with local demand profiles and institutional realities—while supporting them through complementary measures such as appliance access or subsidized tariffs—remains essential for overcoming the trap and advancing equitable, sustainable electrification."

Reviewer Comment 1.8 — Page 12: "Low demand may stem from household energy preferences (e.g., charcoal for cooking in rural areas), or limited purchasing power to acquire the appliances necessary for higher tier electricity usage." Household preferences may play some role, but appliance affordability is critical. In this case, electric cooking typically is expensive – even in urban areas.

Reply: Thanks for this comment. We acknowledge the importance of appliance affordability over household energy preferences, and have revised the sentence on page 18, line 300-303, as follows:

> "On the demand side, low electricity use is driven primarily by households' limited purchasing power to acquire essential appliances for higher-tier electricity [30,31], and secondarily by enduring preferences for traditional fuels such as charcoal [32]."

Reviewer Comment 1.9 — Page 12: "Tariff structures that fail to reflect geographic and socioeconomic heterogeneity can unintentionally worsen these inequities. Therefore, electrification planning must explicitly incorporate local context and variability." I think this is actually a more complicated issue. What is "geographic heterogeneity" aside from socio-economic heterogeneity? If electricity is costlier in one region than another, why cannot a NATIONAL utility arbitrage those differences in a national power market? It may be because the utility is not actually national in its coverage and inter-connectedness. Does it then follow that the utility has geographically heterogeneous markets, or should it invest in becoming fully national?

Reply: We thank the reviewer for this fair point. Our original wording unintentionally conflated geographic with socioeconomic heterogeneity, and we see how this could be read as implying regional utilities or fragmented power markets, which was not our intention. Our focus was not on utility pricing strategies, but rather on the fact that national-level averages can obscure local disparities—whether driven by income, infrastructure availability, or remoteness. To avoid confusion, we have revised the sentence on page 18, line 308-310:

> "The challenge is further compounded by within-country inequality, which creates pockets of deep energy poverty that national-level statistics obscure."

Reviewer Comment 1.10 — Page 12: "Extending services into low-affordability areas without complementary demand stimulation—such as subsidies for connection fees or appliance financing schemes—risks further fiscal strain for utilities, creating a feedback loop of underutilisation and declining investment." This statement would be clearer if the authors referred back to the conclusions reached by comparing tariff structures. Having high energy charges – well above variable costs – is inefficient and a major disincentive for demand expansion. However, high energy charges often are an effort to offset low standing charges put in place to stimulate connection. These in turn undermine utility financial viability, while high energy charges dissuade households from connecting. A better rate design is energy charges in line with variable cost and higher standing charges, potentially accompanied by TARGETED connection subsidies for lower-income households – and along with this, attention to cost-effective non-grid solutions.

Reply: We thank the reviewer for highlighting the need to connect our policy implications back to tariff structures. We fully agree that distorted rate design—low or absent standing charges offset by disproportionately high per-unit energy charges—creates a two-sided problem: it undermines utilities' financial viability while simultaneously disincentivizing households from expanding their usage once connected.

In light of this comment, we revised the passage on pages 18-19 to explicitly foreground how current tariff structures contribute to suppressed demand and fiscal strain. The revised text now makes three points more explicit:

(1) Tariff design often departs from true cost-reflectiveness and systematically shifts the recovery burden onto per-unit charges;

(2) This creates affordability barriers which drive households to restrict usage to very low levels, suppressing demand growth; and

(3) A more sustainable model would better balance cost recovery and affordability by aligning energy charges with variable costs, applying reasonable standing charges, and supporting low-income households with targeted subsidies.

> Page 18-19, line 311-323:
>
> "A central challenge arises from tariff structures that inadvertently reinforce the *grid electrification trap*. In principle, utilities are expected to set cost-reflective tariffs [34]. Yet in practice, attempts to implement cost-reflective tariffs often translate into very low or absent standing charges offset by high per-unit consumption charges—sometimes exceeding the global average (Figure 2). While such structures aim to recover costs, they may render electricity unaffordable even for connected households (Supplementary Table S2). This dynamic helps explain why, in Rwanda, Chad, and Guinea-Bissau, households restrict their usage to T1–2 levels (< 30 kWh/month; Supplementary Table S3), suppressing demand and leaving utilities with chronically low revenues and persistent losses. To avoid this outcome, a more sustainable approach would align energy charges with variable costs, apply reasonable standing charges, and offer targeted electricity subsidies for economically disadvantaged customers."

Inspired by the reviewer's comment, we revised the discussion to better integrate the results with selected country examples. This revised structure provides a more coherent account of the macro-level barriers to electrification and the feedback loops that explain the slow pace of electrification progress in SSA .

- Page 18, line 290-296:

  "Affordability is the first and most visible barrier to grid electrification: around half of SSA's population cannot afford T4 grid electricity, and affordability drops drastically at T5. Specifically, connection rates remain low even within grid-covered areas [9], as many households cannot afford demand-based charges despite subsidized upfront costs [29]. In countries such as Rwanda, Tanzania, and Kenya, the absence of standing charges has not been sufficient to overcome this affordability constraint (see Figure 1 and Supplementary Table S2)"

- Page 18, line 297-308:

  "However, even when households can theoretically afford grid electricity, actual demand often remains far below potential (see Figure 4 and Supplementary Table S3). This gap stems from both demand- and supply-side factors, which are deeply interlinked. On the demand side, low electricity use is driven primarily by households' limited purchasing power to acquire essential appliances for higher-tier electricity [30,31], and secondarily by enduring preferences for traditional fuels such as charcoal [32]. On the supply side, underfunded utilities struggle to recover costs (see Figure 5) and to expand or maintain grid infrastructure. The resulting grid unreliability—manifested in frequent outages, voltage instability, and even large-scale grid collapses [33]—further discourages households from using grid electricity. These two dynamics reinforce each other in a vicious cycle: weak demand undermines utility revenues, while underfunded and unreliable utilities in turn suppress demand."

- Page 19, line 324-339:

  "Against this backdrop, grid expansion needs to be carefully assessed as it is not always practical, nor feasible. Extending services into low-affordability areas without complementary demand stimulation—such as subsidies for connection fees or appliance financing schemes—risks further fiscal strain for utilities, creating a feedback loop of underutilization and declining investment (*grid electrification trap* quadrant, in Figure 5). Governments should therefore give careful consideration to when and where grid electrification is viable under current tariff structures, as our analysis has shown. In many cases—though not the primary focus of this paper—off-grid solutions may prove more viable. Such solutions have already been successfully integrated in several SSA countries, including Tanzania, Mali, and Ghana [35,36]. The success of electrification strategies should be judged not by the number of connections but by the affordability and reliability of the energy services delivered. Aligning interventions with local demand profiles and institutional realities—while supporting them through complementary measures such as appliance access or subsidized tariffs—remains essential for overcoming the trap and advancing equitable, sustainable electrification."

Response to Reviewer 2

Reviewer Comment 2.1 — This work is highly significant to the fields of energy access, development economics, and sustainable development. It builds upon established literature concerning electricity access in SSA (e.g., Blimpo et al., 2020; Winkler et al., 2011). Some additional and updated references, such as Afrobarometer 9april24-1.pdf instead of the 2022 Afrobarometer, would provide a stronger background. It is also important to include established literature on affordability for electricity in SSA, for instance, "Mapping of affordability levels for photovoltaic-based electricity generation in the solar belt of sub-Saharan Africa, East Asia and South Asia" (Nature-Scientific Reports, 2021).

A few times, grey literature is used instead of peer-reviewed papers. A few examples:

- The cited situation of under-grid connection in Kenya from K Lee et al., "Electrification for "Under Grid" households in Rural Kenya, Development Engineering, Volume 1, 2016, 26-35" states: "For example, in Kenya, a study showed that although the electricity grid covered 80% of households in the surveyed areas, only 50% had a physical connection".
- On line 45, a news on a master's thesis on gender is cited to justify the assumption that "T4 is assumed to be the lowest demand level that would justify grid expansion as opposed to off-grid electrification". It is highly recommended to strengthen this assumption with more robust existing literature.
- On Line 30, the statement "Even worse, arbitrary or unsubstantiated tariff-setting can drive high-consuming customers off-grid (e.g., as seen by South Africa's Eskom)" refers to the "positive" move in South Africa to PV caused by increasing instability of the grid. The growth of solar is a key reason why there has been less load-shedding in 2024.

Reply: Thanks for pointing out these opportunities to update our references and minimize reliance on grey literature. To address this, we have made the following revisions:

- **Updated Afrobarometer Data**
  We have incorporated the latest Afrobarometer data (April 2024) to replace the earlier 2022 version. The manuscript on page 2, lines 35-37, now reads:

  > "For example, in Kenya, a study showed that although the electricity grid covered 74% of households in the surveyed areas, only 54% had a physical connection [9]. This gap is even more pronounced in rural areas [10]."

- **Strengthened References**
  We carefully reviewed all references and replaced weaker sources with more robust literature where possible.

  (1) The previous citation *Private firms are driving a revolution in solar power in Africa* has been removed, as the main text sentence changed.

  (2) The citation of a master's thesis on gendered effects of electrification has been replaced with the World Bank ESMAP Technical Report *Beyond Connections: Energy Access Redefined*, which is widely cited and authoritative.

(3) The previous news sources (*Explainer: Why Nigeria's power grid is failing.* and *Nigeria's power supply woes continue.*) have been replaced with peer-reviewed studies *Addressing Nigeria's electricity challenges: Past, Present, and Future Strategies* and *Voltage collapse in Nigeria power system – Causes and Remedies.*

(4) The reference to *Power Triangle* from an open-source educational site has been removed, since the formula is a basic concept in electrical engineering and does not require citation.

(5) The non–peer reviewed policy brief *Access to reliable and affordable electricity for all Kenyans – and how gender matters* has been replaced with the peer-reviewed article *Recognition of and response to energy poverty in the United States*, which provides a stronger academic foundation for the widely adopted energy poverty line.

(6) The news article *The ZiG: Zimbabwe rolls out world's newest currency. Here's what to know.* has been retained for context, but supplemented with the peer-reviewed study *"Exploring public trust in Zimbabwe gold backed currency (ZiG)"*.

For the few remaining non–peer reviewed references, we have ensured that they come exclusively from credible and authoritative institutions such as the World Bank, International Energy Agency (IEA), and national government or utility datasets. These sources provide official data that are not available in peer-reviewed journals, and we have clarified their use in the Methods.

Reviewer Comment 2.2 — Lack of Granular Contextualization: While the paper provides interesting findings at a country level, it lacks country-specific contextualization.

Reply: Thanks for raising this important point. We fully agree that more granular, country-specific contextualization would enrich the analysis. The main reason we did not conduct a deeper within country analysis is the lack of consistent and comparable data across SSA (i.e. decentralized or off-grid tariff structures, the distribution of off-grid versus grid users, and the operational situation of private power utilities). In addition, each country differs in its economic conditions, urban–rural structure, and policy context, making horizontal comparisons challenging and increasing the uncertainty of the analysis.

We fully acknowledge the usefulness and importance of detailed country-specific analyses; however, such work would require dedicated, in-depth studies and thus lies beyond the scope of the present paper. Thus, we have clarified that this paper is designed to assess grid electrification and tariff structures across SSA in a standardized and comparable manner. It evaluates theoretical affordability based on average household electricity consumption following the Multi-Tier Framework (MTF). To avoid any misunderstanding of our objectives, we have revised the abstract and introduction to more clearly highlight this focus. We also emphasize that our findings provide a foundation for future work that can incorporate more granular data once such datasets become available.

- Abstract, Page 1-2, line 1-18:

  Grid expansion is seen as a key strategy for increasing electricity access across Sub-Saharan Africa (SSA). However, whether utilities can generate sufficient revenue from customers under current tariffs to support the capital investment for grid expansion is unclear. Assessing

this conundrum, this study compiles and analyzes household electricity tariffs for the 48 countries in SSA categorized by household demand levels within the Multi-Tier Framework and household affordability thresholds set as 5%, 10%, and 15%. Under current tariffs, approximately 608 million people (50%) cannot afford Tier 4 (T4) electricity, rising to 1.05 billion (85%) for full Tier 5 (T5) access under 10% energy poverty threshold. Sensitivity checks at 5% and 15% indicate that while absolute numbers vary widely (38% to 71% for T4; 76% to 97% for T5), overall trends and relative country rankings remain consistent. Moreover, even where higher-tier electricity is affordable, usage remains low. In countries with both low affordability and weak utility, grid expansion appears unfeasible. While, in some instances, targeted policy reforms and tariff adjustments may provide a path forward and offer potential remedies to overcome these challenges, off-grid solutions may be the only viable option."

- Page 3, line 48-58:

  "This is important, as the grid continues to constitute a viable and often indispensable option—not necessarily for last-mile distribution in sparsely populated or remote areas, but particularly in regions characterized by higher population density and greater consumption potential, where economies of scale can be more effectively realized. We focus specifically on grid-based electrification, as most countries continue to regard it as the default strategy to address low access rates, and it is our intention here to evaluate the validity of this approach. Nevertheless, this emphasis should not be interpreted as overlooking the potential and importance of off-grid solutions, which remain critical where grid expansion proves infeasible and may, in such contexts, represent the more effective pathway to universal access."

- Page 3-4, line 62-65:

  "Acknowledging that tariffs are likely to rise as connection rates increase—given the high capital cost of reaching harder-to-serve populations—we use current tariffs to present a maximally optimistic view of affordability."

Reviewer Comment 2.3 — Role of Decentralized Energy and Subsidies: The paper does not adequately discuss the role of decentralized energy solutions in addressing the affordability gap, nor the impact of subsidies on government budgets versus utilities (private/national). While briefly mentioned in the discussion, these critical aspects are not integrated into the methods or supplementary information.

Reply: We agree that decentralized energy solutions and subsidies stargety on utility play a crucial role in addressing affordability gaps and influencing the financial health of utilities. However, as discussed in the response to Comment 1.6, 1.7 and 2.2, the main reason we did not include this types of factor in our methods or conduct an in-depth analysis is the lack of a comparable database, which makes it nearly impossible to perform meaningful analyses. We do acknowledge the importance of these factors, which can be further studied once relevant data become available (e.g., off-grid tariff structures, private utility financial situations). Our current paper can serve as a solid foundation for future comparisons and analyses.

Additionally, we have tightened the focus of the paper to emphasize that our modeling addresses grid electricity tariffs only, and we have clarified that our discussion adopts a macroscopic perspective, identifying contexts where grid expansion could be theoretically feasible based on affordability and the financial health of utilities. From the results and discussion, we conclude that most regions in SSA located in quadrants B, C, and D are more viable for off-grid solutions, while in some cases, such as rural areas of Gabon (quadrant A), grid expansion is also infeasible. Therefore, although we did not conduct a quantitative analysis of off-grid electrification strategies, we do recognize their importance and have incorporated this discussion into the main manuscript, as follows:

> Page 14-16, line 228-253:
>
> "First, countries in the *grid electrification opportunity* quadrant (**A**) have approximately 110 million people who lack access to electricity. Expanding the grid could bring substantial benefits to underserved region. However, it is essential to carefully consider the geographic distribution of uneletrified housholds, even though our analysis indicates that countries in quadrant **A** show high potential for grid expansion. To illustrate, in Gabon (GAB), households spend on average only 3% of their income to afford T4 electricity, while the national utility, Société d'Énergie et d'Eau du Gabon (SEEG), maintains a profit margin of 1.7% relative to revenue. These results suggest that the utility may have sufficient financial capacity to invest in grid expansion, and that households are likely able to afford electricity at the current tariff level. However, by 2023, although Gabon had achieved a national electrification rate of 94%[24], rural areas lagged significantly behind, with only 29% coverage[25]. Rural residents are widely dispersed across tropical rainforests and hilly terrains, whereas power generation and transmission systems are concentrated in coastal and central cities such as Libreville, Port-Gentil, and Franceville. This spatial mismatch reduces the economic viability of grid expansion to remote rural areas. A significant rural–urban electrification disparity is also observed in Ethiopia (ETH) and Tanzania (TZA)[24,25,26]. These cases demonstrate that utilities' overall affordability and profitability outcomes are largely driven by urban households. To achieve universal energy access, greater attention should be given to rural areas, with careful consideration of distance and cost in electrification strategies—particularly in relation to the size of underserved regions and their proximity to the existing grid infrastructure. There is also a possibility that revenues from urban consumers may need to be leveraged to cross-subsidize rural electrification, a dimension that requires further analysis. By contrast, in smaller island states such as Mauritius (MUS) and Seychelles (SYC), grid expansion can more readily achieve last-mile connections."

Reviewer Comment 2.4 — Affordability Calculation Granularity: The affordability calculations, particularly the "Ten Percent Rule," use national average data for income expenditure rather than specific income tiers for households. This overlooks potential differences between rural/urban populations or those close/far from the grid. As highlighted, lower-income households consistently spend a significantly higher percentage of their income on energy, and a national average might obscure the true extent of affordability challenges for vulnerable groups.

Reply: We thank the reviewer for raising this important point. Our response has two points:

(1) **Affordability thresholds**

As noted in response to Reviewer 1, Comment 1.3, the 10% threshold is widely adopted as a benchmark for energy poverty Bednar and Reames [2020], Galvin [2024], L.Taylor [1993], Muhihi [2024], Simshauser [2021], Winkler et al. [2011], Zhang et al. [2019]. We agree that lower-income groups may be willing to spend a higher share of their income on electricity, as their expenditures on other goods are often at subsistence levels. This is closely related to the proportion of disposable income that households can allocate to electricity, which is highly subjective and uncertain. Therefore, it remains necessary to apply a globally accepted standard to analyze the results (i.e 10% energy poverty line).

However, we acknowledge that using a single affordability threshold may not be fully convincing. We therefore conducted a sensitivity analysis using alternative affordability lines: 5% (as applied in the World Bank's affordability analysis) and 15% (which may better reflect rural populations' willingness). The results, presented in Supplementary Table S1, show that the share of households unable to afford electricity varies considerably depending on the threshold chosen, ranging from 71% to 38% in Tier 4 and from 97% to 76% in Tier 5. While the absolute magnitude of affordability stress changes, the country rankings and policy implications remain consistent. This consistency confirms the robustness of our conclusions. We have clarified this sensitivity analysis in the revised Abstract, "Affordability" section and Methods.

- Abstract, Page 1, line 1-18:

  "Grid expansion is seen as a key strategy for increasing electricity access across Sub-Saharan Africa (SSA). However, whether utilities can generate sufficient revenue from customers under current tariffs to support the capital investment for grid expansion is unclear. Assessing this conundrum, this study compiles and analyzes household electricity tariffs for the 48 countries in SSA categorized by household demand levels within the Multi-Tier Framework and household affordability thresholds set as 5%, 10%, and 15%. Under current tariffs, approximately 608 million people (50%) cannot afford Tier 4 (T4) electricity, rising to 1.05 billion (85%) for full Tier 5 (T5) access under 10% energy poverty threshold. Sensitivity checks at 5% and 15% indicate that while absolute numbers vary widely (38% to 71% for T4; 76% to 97% for T5), overall trends and relative country rankings remain consistent. Moreover, even where higher-tier electricity is affordable, usage remains low. In countries with both low affordability and weak utility, grid expansion appears unfeasible. While, in some instances, targeted policy reforms and tariff adjustments may provide a path forward and offer potential remedies to overcome these challenges, off-grid solutions may be the only viable option."

- Page 9, line 138-150:

  "To evaluate the affordability of grid electricity bills, we simulate income distributions for each country and estimate the share of the population that could afford T4 or T5 electricity access under 5%, 10%, and 15% thresholds (see Methods and Supplementary Table S1). In SSA, the share of population unable to afford T4 electricity is approximately 71% at the 5% threshold, 50% at the 10% threshold, and 38% at the 15% threshold. For T5 access, the corresponding unaffordability shares rise to 97%, 85%, and 76%, respectively. The size of the population deemed unable to afford electricity is highly sensitive to the affordability threshold applied, which in turn reflects the share of disposable income that households

at different income levels are willing or able to allocate to electricity. Although absolute values differ, the overall patterns and relative rankings across countries remain consistent across all affordability thresholds. Since the 10% is widely adopted as the benchmark for defining energy poverty, we adopt this as the primary reference in the following analysis."

- Page 9-10, line 158-168:

  "Affordability is especially severe in countries such as Burkina Faso (BFA), Burundi (BDI), the Central African Republic (CAF), Chad (TCD), Comoros (COM), Eritrea (ERI), Guinea-Bissau (GNB), Liberia (LBR), Madagascar (MDG), Mali (MLI), Niger (NER), and Sierra Leone (SLE), where less than 10% of the population can afford electricity across all income thresholds. By contrast, countries such as Angola (AGO), Mauritius (MUS), Seychelles (SYC) demonstrate relatively higher affordability, with over 90% of the population theoretically able to cover the associated electricity costs. The affordability challenge becomes even more severe at the Tier 5 level, where the number of people unable to afford electricity rises to 1.05 billion—86% of the population—and most countries experience sharp declines across all income thresholds."

- Page 11, line 186-191:

  "Furthermore, the choice of affordability threshold strongly influences the estimated size of the unaffordable population, even though broader affordability challenges remain evident. Some countries, such as Guinea, Mauritania, and Nigeria, display relatively high affordability under the 10% and 15% thresholds but experience sharp declines when the stricter 5% threshold is applied."

- Methods, Page 25, line 443-449:

  "We adopt the widely recognized "Ten Percent Rule" as our affordability threshold, which suggests that spending 10% or more of a household's income on electricity indicates energy poverty [54,55]. This criterion has been applied in various international studies [14,15,56,57,58]. To test robustness, we also conduct sensitivity analyses with 5% and 15% thresholds [59,60], confirming that while absolute magnitudes shift, the geographic patterns and policy conclusions remain consistent."

(2) **Geographic distribution**

We acknowledge that our analysis does not identify the precise geographic locations of affected households, as comparable geospatial data on electrified populations are unavailable across SSA countries. Incorporating inconsistent or incomplete datasets could increase the uncertainty of the results. Therefore, our analysis adopts a macroscopic perspective on the viability of grid expansion, for which a standardized, national-level approach is more appropriate and reliable. While detailed spatial data collection lies beyond the scope of this study, it remains an important avenue for future within-country research.

Reviewer Comment 2.5 — Peak Demand Assumptions: The assumptions for Peak Demand for Energy appear to be very rough, using a basic basket of appliances without sufficiently accounting

for wealth divergences and climatic conditions between countries. This could affect the accuracy of the estimated electricity usage tiers.

Reply: Our baseline peak demand values strictly follow the electricity usage levels, current limits, and appliance baskets defined in the MTF. The MTF tiers are designed to capture not only electricity quantities but also appliance ownership, which implicitly incorporates wealth-related differences: lower tiers (T1–T3) exclude high-power appliances that are uncommon in rural and low-income households, while higher tiers (T4–T5) include high-power appliances more typical of wealthier and urban households. For T5, we adopt the MTF definition in full, assuming that all high-power appliances operate simultaneously to represent a maximum plausible peak load under extreme conditions, as detailed in Supplementary Table 4. This approach ensures methodological consistency across tiers and countries while capturing the upper bound of potential demand.

We also acknowledge that climatic conditions may influence peak demand, particularly through heating and cooling, but comparable high-resolution data on climate-driven appliance use and customer preferences are not consistently available across all 48 SSA countries. Attempting to standardize such adjustments without robust data would introduce greater uncertainty. We therefore retain the MTF-based definitions for comparability across the region, while acknowledging that climate impacts remain an important factor for future within-country studies. This consideration is explicitly noted in the Methods.

> Page 21, line 373-375:
>
> "Climatic conditions may also influence this estimate; however, insufficient data introduce additional uncertainty."

References

Dominic J. Bednar and Tony G. Reames. Recognition of and response to energy poverty in the United States. *Nature Energy*, 5(6):432–439, 2020. doi: 10.1038/s41560-020-0582-0.

Fabio Clementi and Mauro Gallegati. *Pareto's Law of Income Distribution: Evidence for Germany, the United Kingdom, and the United States*, pages 3–14. Springer Milan, Milano, 2005. ISBN 978-88-470-0389-7. doi: 10.1007/88-470-0389-X_1. URL https://doi.org/10.1007/88-470-0389-X_1.

Jon X. Eguia and Dimitrios Xefteris. Lognormal (re)distribution: A macrofounded theory of inequality. *European Economic Review*, 170:104863, 2024. ISSN 0014-2921. doi: https://doi.org/10.1016/j.euroecorev.2024.104863. URL https://www.sciencedirect.com/science/article/pii/S0014292124001922.

Ray Galvin. Reducing poverty in the UK to mitigate energy poverty by the 10% and LIHC indicators: What tax changes are needed, and what are the consequences for CO2 emissions? *Ecological Economics*, 217:108055, 2024. ISSN 0921-8009. doi: https://doi.org/10.1016/j.ecolecon.2023.108055. URL https://www.sciencedirect.com/science/article/pii/S092180092300318X.

L.Taylor. Book review: Fuel poverty from cold homes to affordable warmth. *Energy Exploration & Exploitation*, 9(6), 1993. doi: https://doi.org/10.1177/014459879100900626.

Bikolimana Giliadi Muhihi. Affordability of electricity to rural consumers in Tanzania: An elephant in the room? *Interdisciplinary Journal of Rural and Community Studies*, 6, 2024. doi: https://doi.org/10.38140/ijrcs-2024.vol6.02.

Paul Simshauser. Vulnerable households and fuel poverty: Measuring the efficiency of policy targeting in Queensland. *Energy Economics*, 101:105405, 2021. ISSN 0140-9883. doi: https://doi.org/10.1016/j.eneco.2021.105405. URL https://www.sciencedirect.com/science/article/pii/S0140988321003029.

Harald Winkler, André Felipe Simões, Emilio Lèbre la Rovere, Mozaharul Alam, Atiq Rahman, and Stanford Mwakasonda. Access and affordability of electricity in developing countries. *World Development*, 39(6):1037–1050, 2011. ISSN 0305-750X. doi: https://doi.org/10.1016/j.worlddev.2010.02.021. URL https://www.sciencedirect.com/science/article/pii/S0305750X11000623.

Dayong Zhang, Jiajia Li, and Phoumin Han. A multidimensional measure of energy poverty in China and its impacts on health: An empirical study based on the China family panel studies. *Energy Policy*, 131:72–81, 2019. ISSN 0301-4215. doi: https://doi.org/10.1016/j.enpol.2019.04.037. URL https://www.sciencedirect.com/science/article/pii/S0301421519302861.

Second-Round Response to Reviewers in Support of Appeal for Manuscript NCOMMS-25-31084A

Thank you very much to the editor and the reviewers for their time and effort in evaluating our manuscript. Reviewers' comments are shown in black, and our point-by-point responses are provided in blue.

We sincerely appreciate the reviewers' comments; however, several points appear to reflect misunderstandings of the work we conducted in this study. To prevent further confusion and to improve the clarity of the manuscript, we have revised the text accordingly. Newly added information is highlighted in red, while revisions that preserve the original meaning but involve minor rephrasing are shown in gray in the revised manuscript.

**Reviewer Comment**	**Author Response**
**Reviewer #1**	
I appreciate the efforts of the authors in responding to my comments on the previous version of the paper. I have no additional suggestions.	We thank the reviewer for their time and are glad to read that our revisions addressed all points satisfactorily.
**Reviewer #2**	
The paper presents a valuable compilation of country-level data, and this is certainly one of its strengths.	We are glad to read that the reviewer deems the analysis of general interest.

However, the analytical component remains relatively limited. The conclusions on affordability rely primarily on national averages, although energy poverty varies substantially across rural and urban contexts.	We thank the reviewer for the comment, although we think that concerns about the general analytical value of the paper should have been pointed out in the previous round of revisions. In our analysis, we do not calculate national averages for affordability. Instead, we take care to model income distributions based on empirical evidence. As described in the Methods (Equations 5–13), affordability is calculated at the 1-percentile level (p1–p100) based on each percentile's income distribution within a country. We model these distributions at the percentile level because the upper end is difficult to estimate, as noted frequently in the literature [1, 2]. In contrast, the lower end—where accuracy matters most in our study context—can be well-captured by a lognormal specification, according to many previous research papers [3, 4]. The results per percentile are available in related GitHub link.<br><br>We acknowledge that Figure 5 presents a single national-level affordability indicator for each country. This was done because the accompanying utility financial performance data (UPBEAT) are only available at the national scale, and a country-level summary is required for a consistent cross-country comparison. Importantly, however, this visualization choice does not imply that our underlying affordability model relies on national-averages; the model itself remains fully percentile-based and captures within-country income heterogeneity. We have added additional clarification to the manuscript, as outlined below:<br>• Abstract, Page 1:<br>"Affordability is assessed for each income percentile using simulated income distributions."<br>• Introduction, Page 4:<br>"This allows us to identify whether residents can afford to consume electricity even after being connected, the percentage of the population that can reasonably afford each tier, and how affordability constraints may limit achievable demand levels."<br>• Affordability, Page 10:<br>"While the shapes of the distributions differ, the key driver of affordability is the relative position of the T4 and T5 bill amounts within these distributions."

	• Methods, Page 20:<br><br>"Although the modeled distribution inevitably simplifies some within-country heterogeneity, it allows us to capture broad regional patterns and test different income groups' affordability across tiers in a way that is both replicable and robust."
Similarly, the affordability calculations use national tariff levels and TIER consumption tiers, which may not fully capture the considerable heterogeneity in consumption patterns across countries and subnational regions.	This paper is the result of an extensive and comprehensive collection of electricity tariff data across sub-Saharan Africa (SSA). While we would welcome evidence of any we have missed, we are confident that our tariff data is extremely comprehensive. We are not aware of any other data source, report or peer-reviewed paper that has collected this data. Collecting this data is not trivial because it is dispersed across different Ministries and published in different languages. We use tariff schedules in effect as of 15 November 2024 for 38 countries, collected from the official national power utility websites, following the same approach adopted by the World Bank's *Making Power Affordable for Africa and Viable for Its Utilities* study [5]. To expand the dataset, we obtain tariff information for the remaining countries from reliable third-party sources (see detailed documentation in the Zenodo). Importantly, we do not simply rely on a single national tariff. Household electricity bills are calculated based on the lowest applicable tariff at each consumption level. When multiple tariff schedules exist, we take their average. All calculation procedures for each country and each tier are documented in detail in the 'electricity bill' Excel file on Zenodo. In addition, we provide SI Table S1 to summarize these different tariff types. We have clarified this point more specifically in the revised manuscript, as follows:<br><br>• Methods, Page 22–23:<br><br>"Therefore, electricity costs for different MTF tiers are calculated using the lowest applicable tariff at each consumption level, following the two approaches described above. When multiple tariff schedules exist, their average is used. All calculation procedures for each country and each tier are documented in detail in the 'electricity bill' Excel file available on Zenodo."<br><br>• Methods, Page 21:<br><br>"Residential tariff structures in effect as of 15 November 2024 were available from official power-utility documents for 38 of these countries."

- Methods, Page 21:

  "Electricity tariff structures differ across countries. Most use social/lifeline tariffs and standard tariffs with either linear or increasing-block pricing for residents, based on metrics such as electricity usage (kWh), active power (kW), apparent power (kVA), duration (hours), and current (A). Namibia (NAM) is the only country identified with clearly defined pensioner tariffs separate from its social tariff, shown on SI Table S1."

- Methods, Page 21:

  "Because households-level demand data are often unreliable or even unavailable across most of SSA [50, 51], we use MTF to estimate potential electricity costs for each energy tier based on current residential tariffs."

- Methods, Page 22-23:

  "Therefore, electricity costs for different MTF tiers are calculated using the lowest applicable tariff at each consumption level, following the two approaches described above. When multiple tariff schedules exist, their average is used. All calculation procedures for each country and each tier are documented in detail in the "electricity bill" Excel file available on Zenodo."

  - **Usage-based method.** For tariffs determined solely by electricity consumption (kWh), the cost is calculated using the minimum electricity usage required for each MTF tier.
  - **Capacity-based method.** For tariff structures that include capacity restrictions—defined by active power (kW), apparent power (kVA), duration (hours), or current (A) for differentiating domestic customer categories—monthly charges based on maximum capacity (A, kVA, or kW) are incorporated together with the minimum usage (kWh).

  The electricity cost calculation is based on the following assumptions:

  - **Connection type.** Only single-phase residents' connections are considered, as they represent the standard configuration in most countries, while three-phase connections are primarily used in commercial and industrial settings. Although some countries (e.g., ZAF [56,57] offer three-phase domestic tariffs, residents adoption remains limited.

	– **Tier scope.** We focus on grid electricity costs for T4, with T3 and T5 as low and high end sensitivity cases, as this approach is more suitable for assessing the potential for grid expansion and providing long-term guidance toward achieving SDG7. For T1 & 2, alternative off-grid solutions (e.g., solar PV, diesel generators) are generally more practical and effective.<br>– **Climatic influence.** Climatic conditions may affect electricity consumption patterns; however, no data are currently available for this purpose.<br>– **Currency and year standardisation.** All electricity bill data, reported in various currencies and years, are converted to 2024 US dollars using Eq. 3-4:<br><br>Similarly, there is simply no comprehensive demand data available for SSA, let alone differentiated by subnational regions. We therefore use Tiers to represent different demand levels and show the resulting electricity cost by Tier. As such, we provide maximum transparency on potential electrification costs and we are not aware of any data source or method that would enable a more fine-grained analysis across the continent. Recent assessments corroborate this data scarcity: the African Climate Foundation's report *Energy Data Challenges in Africa's Electricity Landscape* shows that only South Africa has near-real-time, publicly available demand data, while more than 40% of African countries have only outdated national data or none at all [6]. In addition, EMBER's evaluation of electricity-data transparency across Africa assigns most countries a data score of just 0–1 (5 is the highest score) [7]. We acknowledge this urgent need for better data in the discussion.<br><br>• Demand expectations, Page 14:<br>"Due to data availability, the analysis is conducted at the country level, and the corresponding energy tiers are identified accordingly."<br><br>• Discussion, Page 19-20:<br>"This underscores the need for more granular, subnational data—particularly at the district or county level—to accurately diagnose localized constraints and design interventions that account for spatial heterogeneity in demand, affordability, and service reliability."

As a result, the analysis does not fully reflect the complexity of affordability dynamics or their spatial disparities.	We agree that there are further complexities in affordability levels and that spatial distribution is a crucial one. However, we intend to provide a Continent-wide data set on electricity tariffs in this paper, and there is not data to dive deeper into these complexities. Indeed, for most of the studied countries, this would even be impossible at the national level. As shown in recent work in Nature Energy, national electrification statistics in many African countries exhibit substantial inconsistencies [8]. Complementary assessments by the African Climate Foundation and Ember further demonstrate that most countries lack reliable national-level data and almost none publish harmonised sub-national or high-frequency demand information [6, 7]. As such, it is effectively impossible to undertake a highly-resolved spatial analysis of affordability dynamics which is comprehensive throughout SSA using existing data. The IEA also explicitly highlights these structural data gaps [9]. Given these well-documented limitations, we think that a critique because of an alleged lack of complexity is unfair.
As an example, Figure 2 provides a useful high-level overview of tariff variations by grouping countries into four categories; however, the distinctions among social, domestic, pensioner, and general tariffs are not easily interpretable in the current format.	We appreciate the reviewer's comment regarding the interpretability of the tariff categories in Figure 2. To improve readability, we provide a more detailed explanation in the figure caption and align it with the tariff-structure summary presented for each country in SI Table S1.<br><br>• Grid tariffs and electricity bills, Page 5:<br>"Figure 2 illustrates how the average unit price ($/kWh) varies with usage across countries under current different residential tariff types, revealing four distinct patterns (Groups I–IV). Countries in Group I (15 countries) apply standing charges that are independent of usage levels. These fixed costs cause high average costs at low usage levels (T1 to T2), disproportionately affecting low-usage and lower-income households. This regressive effect is particularly evident in eight countries within this group—such as Benin (BEN), BFA, and Côte d'Ivoire111 (CIV)—where a separate social or lifeline tariff is nominally provided for low- income. and vulnerable consumers. In practice, however, residents who typically use less than 30 kWh per month still face a higher effective cost per kWh than those on standard tariffs, because standing charges remain in place even under the social tariff, thereby further increasing the burden on vulnerable residents."

- Grid tariffs and electricity bills, Page 7:

  "The steepness varies widely, reflecting differences in how countries define their block thresholds and corresponding prices. Although most countries in this group—aside from Kenya and Uganda (UGA)—do not have explicit lifeline or social tariffs, the IBT structure itself provides lower effective prices at minimal consumption levels, thereby offering some protection for low-usage residents. When block increments are relatively moderate, residents can move into higher usage levels more smoothly; however, steep marginal prices can make additional consumption increasingly unaffordable, suppressing demand and, in some cases, prompting residents to exit the grid altogether."

- Figure 2 Caption, Page 9:

  "Countries are grouped into four categories (I–IV) based on visible differences in how their tariff structures translate into effective unit prices as usage increases. The cost per kWh in each plot is calculated strictly from the country's various residential tariff schedules—using the applicable blocks, standing charges, and unit rates—and dividing the resulting monthly bill by the corresponding consumption level. The background shading reflects the MTF energy tiers (T1 to T5); these tiers are shown only to contextualize where each consumption point lies within the MTF framework."

The cost per kWh shown in the figure is derived by dividing the monthly residential electricity expenditure—calculated from the tariff schedules in effect as of 15 November 2024—by the corresponding consumption level. Each colored panel represents a different energy tier, indicating the consumption range and the associated effective unit cost of electricity within that tier.

In practice, the vast majority of utilities do not differentiate tariffs by socio-demographic attributes such as pensioner vs. working-age households or domestic vs. foreign residents. Based on our dataset of 48 countries, only Namibia apply specific pensioner tariff, and none apply different tariffs for foreign and domestic households.

The tariff schedules in effect as of 15 November 2024 indicate that 19 countries (approximately 40%) clearly define social or lifeline tariffs. For these countries, we calculate the electricity bill separately based on their restricted amount of consumption, see SI Table S1.

The discussion could be strengthened by incorporating the influence of energy policy and regulatory frameworks—such as different subsidy schemes—on affordability outcomes.	We thank the reviewer for this remark. The intention of our paper is not to calculate affordability in detail. Rather, it is to cast light on the likeliness of grid-connected electrification across Africa under existing institutional and pricing frameworks. Naturally, national governments can decide to subsidize grid-connected access to the extent they wish and this would make such forms of access more affordable to the population. Since any existing subsidies are already embedded in these tariffs, the data we use inherently reflect the influence of the current regulatory framework and subsidy schemes on price structures, as well as their financial implications for utilities. Consequently, the effects of the prevailing policy environment on both consumer expenditure and utility-level financial pressure are already implicitly captured in our analysis. However, we intend to contrast grid-connected electrification with other forms of electrification and for this comparison, it makes sense to leave out any potential future subsidies because, naturally, governments could also decide to subsidize other forms of electrification. Moreover, it can be argued that given current levels of indebtedness that many African countries face, large-scale subsidy programmes to lessen the cost of electrification will be difficult to sustain. This is also reflected in Figure 5, where we suggest that grid-based electrification is more likely feasible in situations where the financial health of the utility is sound. We therefore think that we provide reasonable context and discussion for our results and incorporating additional subsidies would not strengthen our argument.
At present, the findings mainly confirm broad regional patterns across TIER levels, with affordability being closely aligned with the poverty proxy used.	We thank the reviewer for raising this important point. We understand the concern that our affordability patterns may appear closely aligned with the poverty proxy. We also acknowledge that our earlier wording—stating that income differences overwhelmingly drive cross-country affordability—may have unintentionally suggested that tariffs play little or no role in shaping affordability outcomes. This was not our intention.<br><br>Our revised manuscript now clarifies that income and tariffs jointly determine electricity affordability, but they do so through different mechanisms and at different points of the income distribution. Cross-country variation in affordability is indeed strongly influenced by income disparities, reflecting the broader economic capacity of each population. However, this does not imply that tariffs lack meaningful influence. On the contrary, when affordability is examined across the income distribution, rather than at country averages, tariff levels exert their strongest effects at the bottom of the income distribution, where affordability constraints are most acute. Even small tariff differences can fully exclude low-income households from higher-tier electricity use.

As an example, in Angola and Comoros, the bottom 10% of the population earns approximately the same income (about USD 32 per month). Yet the Tier 4 electricity cost is USD 1 in Angola versus USD 52 in Comoros. As a result, a person at the 10th income percentile would spend only 3.5% of their income on T4 electricity in Angola, compared with an impossible 162.6% in Comoros. Here, income is nearly identical, and tariff differences alone determine affordability outcomes. This demonstrates that the poverty proxy cannot explain the observed variation; tariffs play a decisive role for low-income groups.

This is precisely why our income-distribution approach is essential. Rather than reproducing poverty patterns, it reveals where in the income distribution tariff levels push electricity access beyond reach—insights that would be invisible from income data alone. We have revised the manuscript accordingly to make this interpretation explicit and to avoid any misunderstanding that tariffs are unimportant.

- Affordability, Page 10:

  " While the shapes of the distributions differ, the key driver of affordability is the relative position of the T4 and T5 bill amounts within these distributions."

- Affordability, Page 11:

  "Cross-country differences in affordability are shaped by both income and electricity tariffs, although income disparities account for much of the variation observed across SSA."

- Affordability, Page 11-12:

  "Conversely, countries with comparable low-income levels can display very different affordability outcomes when tariff levels diverge. For example, AGO and COM have similar incomes among the lowest decile, but COM's T4 bill ($52) is more than fifty times higher than AGO's ($1), shown in Figure 1, rendering T4 entirely unaffordable for low-income residents in COM while easily attainable in AGO. These contrasts show that tariff levels exert their strongest influence at the bottom of the income distribution, where even small price differences can fully exclude low-income residents from higher energy tiers. Overall, a country's overall affordability profile is primarily determined by the population's broader economic capacity, whereas affordability across different income groups depends largely on tariff setting."

	"This asymmetry is further reflected in the Group I countries in Figure 2: aside from MUS, standing charges raise the effective unit price of very low consumption (T1 and T2) to levels that are already unaffordable for most low-income residents. Although the unit price declines as consumption increases to T4 and T5—a pattern observed across all tariff structures—affordability simulations indicate that these residents remain unable to afford the resulting total monthly electricity cost, even under optimistic assumptions. In practice, low-income users are therefore caught between prohibitively high unit prices at minimal usage and unaffordable absolute bills at higher consumption levels, rendering grid electricity effectively out of reach. Consequently, despite the presence of nominal social tariffs in several countries, prevailing tariff structures fail to provide meaningful protection for low-income users and do not support their progression beyond minimal levels of electricity access."<br>• Discussion, Page 19: "Taken together, affordability challenges arise not only from low incomes but also from tariff structures that systematically limit the ability of low-income residents to progress beyond minimal electricity consumption. Even where social/lifeline tariffs exist, prevailing tariff designs often fail to achieve actual affordability, both at current levels of electricity use and in enabling sustained progression to higher consumption levels. This disconnect underscore the urgent need for several SSA countries—particularly those in Group I shown in Figure 2—to reassess the design and targeting, ensuring that cost recovery is achieved without constraining low-income residents to persistently low levels of electricity consumption."

In summary, while the datasets are of clear value and the topic is important, the manuscript may be better suited to a specialized journal where data-driven analyses of energy access are a central focus for the readership. I hope the authors find this feedback constructive and helpful in refining their work further for another Journal.	We are encouraged to read that the reviewer finds the dataset of value and the topic of importance. Research on African countries, and developing countries in general, is always complicated by a lack of granular data. We believe that we do what is possible given data availability in this paper. We therefore believe that the paper would be of general interest given the relevance of the topic and the novelty of the data. In any case, if the reviewer is aware of datasets which would enable a more granular analysis, we would be happy to consider these.

References

[1] John Sabelhaus, David Johnson, Stephen Ash, David Swanson, Thesia Garner, John Greenlees, and Steve Henderson. Is the consumer expenditure survey representative by income? Working Paper 19589, National Bureau of Economic Research, October 2013.

[2] Angus Deaton. Measuring poverty in a growing world (or measuring growth in a poor world). *The Review of Economics and Statistics*, 87(1):1–19, 02 2005.

[3] Jakub Nedvěd. The Use of the Lognormal Distribution in Analysing Incomes. *International Days of Statistics and Economics: Conference Proceedings (22–23 September)*, ed. Tomáš Löster and Tomáš Pavelka, vol. 5, pp. 451–463. Available at: `https://msed.vse.cz/files/2011/Nedved.pdf`, 2011.

[4] Fabio Clementi and Mauro Gallegati. *Pareto's Law of Income Distribution: Evidence for Germany, the United Kingdom, and the United States*, pages 3–14. Springer Milan, Milano, 2005.

[5] Masami Kojima and Chris Trimble. *Making Power Affordable for Africa and Viable for Its Utilities*. World Bank, Washington, DC, 2016. License: CC BY 3.0 IGO.

[6] African Climate Foundation. Energy data challenges in africa's electricity landscape. Online, Mar 2024. Accessed: 2025-12-02.

[7] Ember Climate Group. African electricity data transparency. Online report, 2022. Accessed: 2025-12-02.

[8] Stephanie Hirmer, Julia Tomei, Pu Yang, Alycia Leonard, Philipp Trotter, Ariane Millot, Florian Egli, Koen van Dam, Agnese Beltramo, and Martin Stringer. Inconsistent measurement calls into question progress on electrification in Sub-Saharan Africa. *Nature Energy 9*, page 1046–1050, 2024.

[9] International Energy Agency. Data-driven electrification in africa. Online programme page, 2024. Accessed: 2025-12-02.
